# Investigating Communication Dynamics in Neuronal Network using 3D Gold Microelectrode Arrays

**DOI:** 10.1021/acsnano.4c03983

**Published:** 2024-06-20

**Authors:** Kui Zhang, Yu Deng, Yaoyao Liu, Jinping Luo, Andrew Glidle, Jonathan M. Cooper, Shihong Xu, Yan Yang, Shiya Lv, Zhaojie Xu, Yirong Wu, Longzhe Sha, Qi Xu, Huabing Yin, Xinxia Cai

**Affiliations:** † State Key Laboratory of Transducer Technology, Aerospace Information Research Institute,, Chinese Academy of Sciences, Beijing 100190, China; ‡ School of Electronic, Electrical and Communication Engineering, University of Chinese Academy of Sciences, Beijing 100049, China; § State Key Laboratory of Medical Molecular Biology, Institute of Basic Medical Sciences, Chinese Academy of Medical Sciences and Peking Union Medical College, Beijing 100005, China; ∥ James Watt School of Engineering, University of Glasgow, Glasgow G12 8LT, United Kingdom

**Keywords:** *in vitro* neuronal network, three-dimensional microelectrode arrays, neuronal network dynamics, synaptic delay, mutual information network, network communication speed

## Abstract

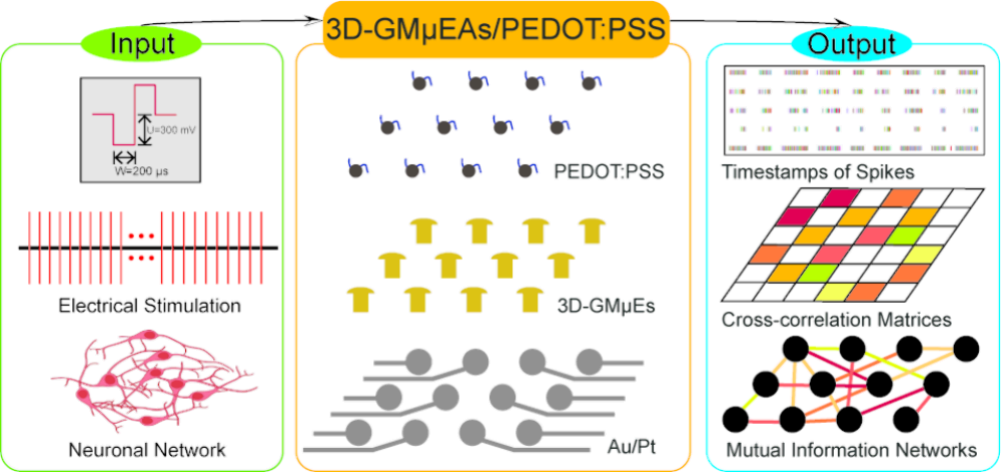

Although *in vitro* neuronal network models hold great potential for advancing neuroscience research, with the capacity to provide fundamental insights into mechanisms underlying neuronal functions, the dynamics of cell communication within such networks remain poorly understood. Here, we develop a customizable, polymer modified three-dimensional gold microelectrode array with sufficient stability for high signal-to-noise, long-term, neuronal recording of cultured networks. By using directed spatial and temporal patterns of electrical stimulation of cells to explore synaptic-based communication, we monitored cell network dynamics over 3 weeks, quantifying communication capability using correlation heatmaps and mutual information networks. Analysis of synaptic delay and signal speed between cells enabled us to establish a communication connectivity model. We anticipate that our discoveries of the dynamic changes in communication across the neuronal network will provide a valuable tool for future studies in understanding health and disease as well as in developing effective platforms for evaluating therapies.

*In vitro*, neuronal network models offer well-known advantages in studying fundamental mechanisms of brain function, including their high controllability and good repeatability, providing information that cannot be obtained from *in vivo* experiments.^1−3^ Consequently, they have become indispensable tools in neuroscience research, facilitating investigations of functional connections between cells, information transferring, underlying mechanisms of brain dysfunction and the impacts of neurological drugs.^4−6^ Among these tools, cell monitoring technologies have become increasingly important due to a rising demand for deeper insights into cellular dynamics for disease research and therapeutic development.^7,8^

It is already well-known that communication dynamics are closely associated with neuronal network function. However, the understanding of the dynamics of cell-to-cell communication during neuronal network development and maturation has been hindered by the lack of sensors with a necessarily high signal-to-noise ratio (SNR) and stability to make long-term recordings, thereby limiting the potential application of *in vitro* neuronal network models for studying brain diseases and neuroscience research, in general.

One commonly used tool for monitoring *in vitro* neuronal networks is the planar, two-dimensional (2D), microelectrode array (MEA), which can monitor of the activity of multiple neurons with high temporal and spatial resolution.^9−12^ However, currently such planar 2D MEAs exhibit a low SNR due to poor coupling between the electrode surface and electrically active cell membrane. This results in only about 1% of the raw evoked signals actually being recorded.^13−15^ Thus, while planar MEAs still serve as a reference benchmark, they are not the ideal tool for studying neuronal networks.^16,17^

3D MEAs have provided a promising alternative to overcome the limitations of planar MEAs.^18−20^ Not only is the electrical conductivity improved, but the biocompatibility of a textured 3D structure can increase the adhesion between the electrode and the neurons, thus reducing the area of electrodes exposed to the electrolyte. The incomplete surface coverage and signal “noise” arising from this exposed area is one of the main reasons for the poor SNR in planar MEAs. In addition, 3D structures can expand the active surface area of the electrodes, giving rise to decreased electrode impedance and increased monitoring sensitivity.

Various types of 3D MEAs have previously been developed. Early methods included protruding or spiked 3D-MEAs,^21^ laser-scribing and electroplating,^22^ vapor–liquid–solid growth of silicon probes,^23^ and DRIE-based processes.^24^ More recent methods have included Metal Transfer Micromolding,^25^ transparent 3D-MEAs,^26^ vertically aligned ultradense carbon nanotube (VACNT)-based 3D-MEAs,^27^ and sputter coating of 3D SU8 structures to create pillar-like electrodes,^28^ volcano-shaped electrodes,^29,30^ and vertically standing nanowire electrodes.^31,32^ While these schemes have collectively improved the SNR for neural signal detection, there is evidence that some of these approaches may cause damage to the cells’ functional membranes and/or have topographical features that prevent cells from adhering. Additionally, the manufacturing process of these 3D MEAs is often complicated, making them difficult to customize as electrode arrays, and thus do not fully meet the criteria for long-term monitoring and regulating dynamic communication within neuronal networks.

As shown in Figure 1, we introduce an electrode fabrication method utilizing PEDOT:PSS-modified 3D gold microelectrode array (3D-GMμEAs/PEDOT:PSS). Our approach involves using planar MEA as a substrate and through a process involving a highly controlled electroplating process, thereby creating 3D gold electrodes (3D-GMμEs). These electrodes allow reliable, long-term recording of neural activity without causing cellular damage, with its geometry promoting the coupling of electrodes with cell membranes.^33−35^

**Figure 1:**
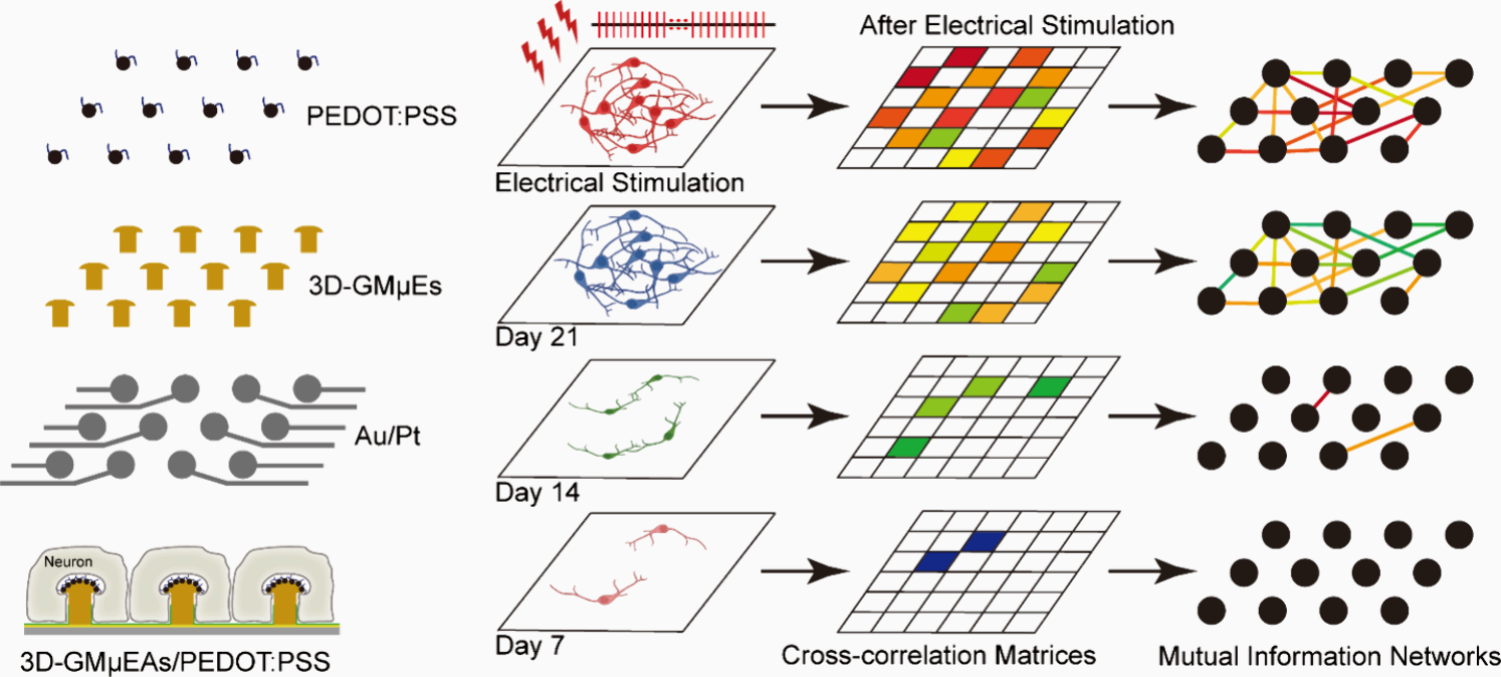
Diagram illustrating the 3D-GMμEAs/PEDOT:PSS and varying communication levels within a neuronal network during a long-term recording spanning 3 weeks.

The conducting polymer PEDOT:PSS is a biocompatible material widely used for neural interfaces which also offers excellent electrical stimulation properties.^36−38^ By modifying the 3D-GMμEAs with PEDOT:PSS provides an array that is not only easy to fabricate in a reproducible manner, but which has outstanding electrical properties and biocompatibility, enabling long-term neuron culture and neural information recording. The same electrode array can be used for directed local electrical stimulation, enabling us to spatially and temporally regulate the network. Furthermore, the electrode arrays can be easily customized to meet different experimental requirements.

Using these sensors, we successfully monitored the dynamic changes in communication activities in the neuronal network over 3 weeks, measuring the synaptic delay between neurons and cell–cell communication speeds across the neuronal network. Using this information, the correlation and mutual information between neurons, we established a method for assessing the communication capability of neuronal networks, using the experimental data to establish a communication connectivity model for neuronal networks at different developmental stages (Figure 1).

## Results and Discussion

### Morphological and Electrical Characteristics of MEA

**Figure 2:**
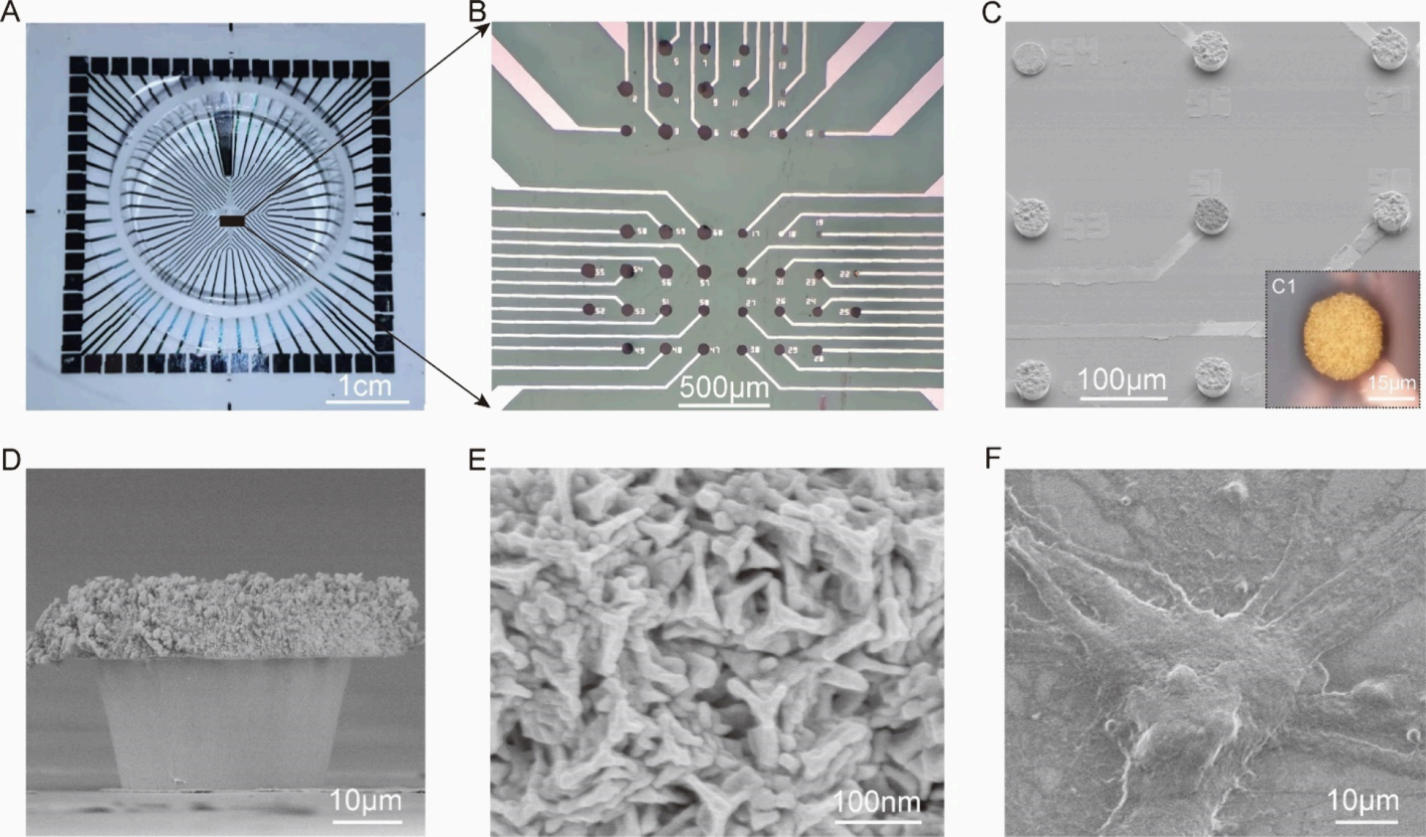
Morphological characteristics of MEA. (A) Micrographs showing completed MEA with a specially designed glass “cloning” ring. (B) Overall view of electrode arrays of different dimensions in Figure 2A. (C) SEM image of part of the 3D-GMμEs array. (C1) Magnified view of a single 3D-GMμE under an optical microscope. (D) SEM image of a single 3D-GMμE in side view. (E) SEM image of 3D-GMμE surface. (F) SEM image of the GMuEs/PEDOT:PSS enveloped by a neuron cell.

Figure 2A show an optical micrograph of the fabricated device with a specially designed glass cloning ring adhered for neuronal culture. An overall view of electrodes in Figure 2A is shown in Figure 2B, where the black color on the electrode surface is the PEDOT:PSS modified electrode. By controlling the plating time, we controlled the growth and thickness of PEDOT:PSS on the electrodes, Figure S1 A, B. Importantly, we can tune the dimension of individual electrodes on the device as illustrated in Figure 2B. The 3D electrode array comprised a series of 30 μm diameter GMμEs electrodes, Figure 2C1, with 200 μm gaps in between, Figure 2C. Each electrode has smooth sidewalls, Figure 2D, and a rough surface that provides numerous sites for PEDOT:PSS modification, Figure 2E. The PEDOT:PSS modification resulted in a surface with a smooth, densely nanosculptured surface (Supplementary Figure S1C, D). As shown in Figure 2F, such 3D electrodes are readily enveloped by the neurons’ cell body. The relatively dense electrode array allows spatial mapping of extended neuron axons with the microscale spatial resolution, Figure S1E, F. It should be noted, that the morphology of the 3D MEA as well as the various features and properties, can be easily modified/customized in a series of controlled actions (including the electroplating time and the PEDOT:PSS composition) for defined application-specific functions.

**Figure 3:**
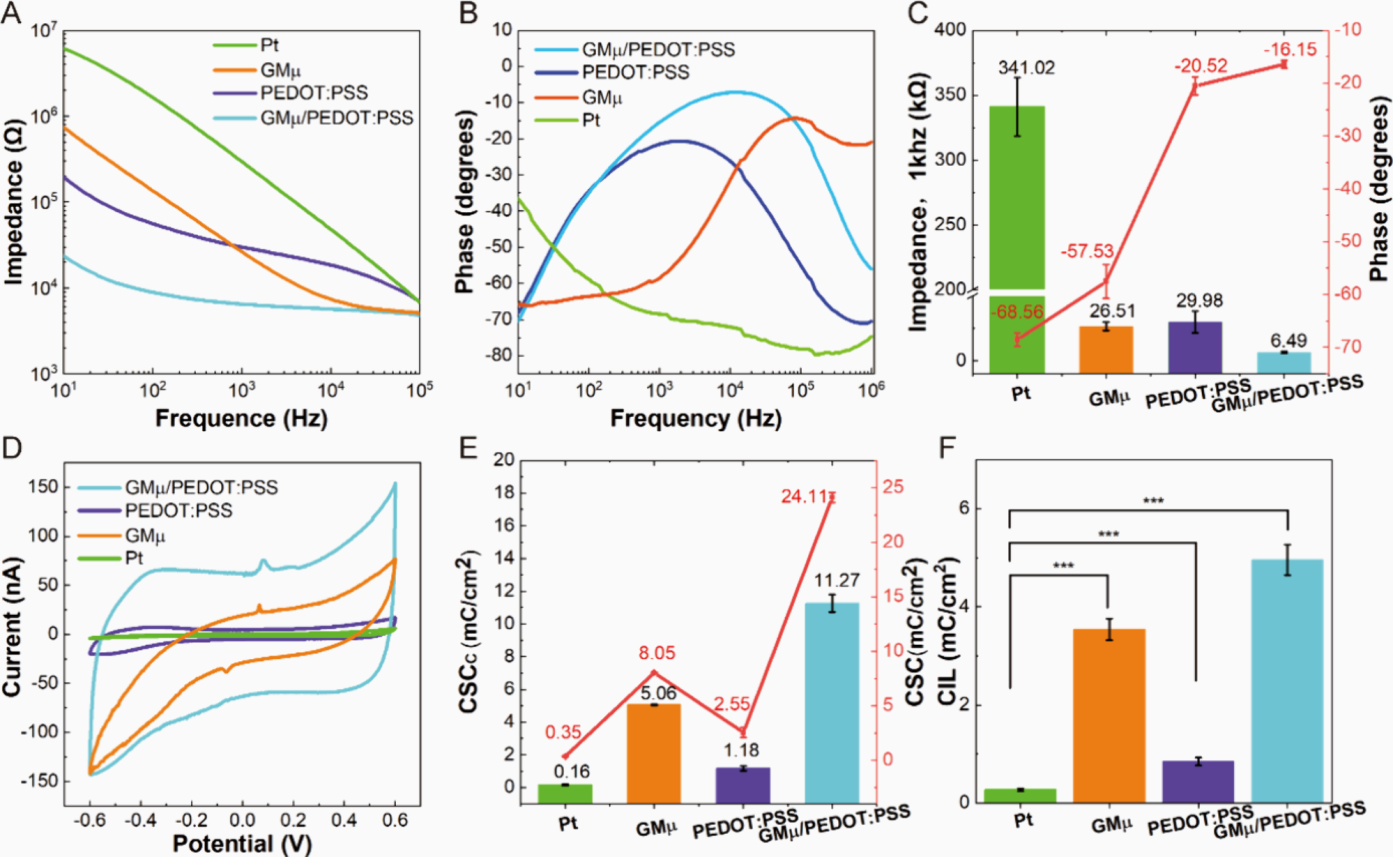
Electrical properties and electrical stimulation of MEA. (A) Impedance properties of bare Pt electrodes, GMμEs, PEDOT:PSS-modified bare Pt electrodes, and GMμEs/PEDOT:PSS at a frequency range of 10 Hz to 0.1 MHz. (B) Phase properties of bare Pt electrodes, GMμEs, PEDOT:PSS-modified bare Pt electrodes, and GMμEs/PEDOT:PSS at a frequency range of 10 Hz to 0.1 MHz. (C) The statistics of impedance and phase at a frequency of 1 kHz, *n* = 5. (D) CV curves of the electrodes scanned in phosphate-buffered saline solution (pH = 7.4). (E) The CSC and CSC_C_ statistics of different types of the devices, *n* = 5 per type. (F) The statistics of CIL tested by VTM, *n* = 5; ***p < 0.001.

To investigate the electrical properties of the electrodes, we conducted electrochemical impedance spectroscopy (EIS) over a range between 10 Hz to 0.1 MHz to measure the impedance and phase shift of various electrodes, including bare platinum electrodes (Pt), GMμEs (GMμ), bare Pt electrodes modified with PEDOT:PSS (PEDOT:PSS) and GMμEs/PEDOT:PSS (GMμ/PEDOT:PSS), Figure 3A, B. We observed a notable reduction in impedance depending on the electrode structure and modification. For example, when exploring electrode EIS at 1 kHz (the frequency selected to monitor neural), we observed that values changed from 341.02 ± 22.40 kΩ for Pt, 26.51 ± 3.24 kΩ for GMμEs, 29.98 ± 8.34 kΩ for PEDOT:PSS, and 6.49 ± 0.67 kΩ for GMμEs/PEDOT:PSS at 1 kHz, Figure 3C, Table S1. Similarly, the phases of the MEAs increased from −68.56 ± 1.21° (Pt) to −57.53 ± 3.24° (GMμ), −20.52 ± 1.71° (PEDOT:PSS), and then increased to −16.40 ± 0.74° (GMμ/PEDOT:PSS), Figure 3C, Table S1. Our results demonstrate that at 1 kHz, the EIS of the GMμEs/PEDOT:PSS has an optimal electrical performance with the lowest impedance and the smallest phase delay compared to other electrodes.

The active surface areas of the GMμEs/PEDOT:PSS (C_dl_ = 1.84 μF) were found to be larger than those of GMμEs (C_dl_ = 0.76 μF) and PEDOT:PSS-modified bare Pt electrodes (C_dl_ = 0.20 μF), indicating that the improved performance is attributed to the increased active surface area, Figure S2. Notably, the phases of the GMμEs/PEDOT:PSS and the PEDOT:PSS-modified bare Pt electrodes were similar, suggesting that the reduction in phase delay is primarily due to the PEDOT:PSS modification. In summary, our findings demonstrate that the 3D-GMμEAs/PEDOT:PSS possess superior electrical properties compared to other electrode types and are well-suited for neural activity monitoring.

### Electrical Stimulation of MEA

The electrical performance of electrodes is known to be a critical factor for the stimulation of neuronal networks. To evaluate the electrodes’ suitability for studying neuronal networks, we characterized their electrical stimulation performance using cyclic voltammetry (CV) and voltage transient measurements (VTM). These measurements allowed us to evaluate the charge storage capacity (CSC) and cathode charge storage capacity (CSC_C_) of the electrodes, as well as their charge injection limit (CIL).

Normally, integration of the CV curve (within the window where hydrolysis is not occurring) is proportional to the CSC, Figure 3D. We found that the CSC of bare Pt electrodes was only 0.35 ± 0.03 mC/cm^2^, while the CSC of GMμEs, PEDOT:PSS-modified bare Pt electrodes, and GMμEs/PEDOT:PSS increased to 8.05 ± 0.23 mC/cm^2^, 2.55 ± 0.31 mC/cm^2^, and 24.11 ± 0.46 mC/cm^2^, Figure 3E, Table S1, respectively. A higher CSC_C_ indicates electrodes with low polarization and high charging capacity characteristics. We found that the CSC_C_ of the MEAs increased from 0.16 ± 0.04 mC/cm^2^ (Pt) to 1.18 ± 0.15 mC/cm^2^ (PEDOT:PSS), then to 5.05 ± 0.04 mC/cm^2^ (GMμ), and finally to 11.26 ± 0.52 mC/cm^2^ (GMμ/PEDOT:PSS), Figure 3E, Table S1. The 3D-GMμEAs/PEDOT:PSS electrodes demonstrated the highest CSC and CSC_C_ values, likely due to their increased specific surface area, which provides a large effective active area for the interaction between the conducting polymer and the surrounding electrolyte.

The CIL is another critical parameter for MEAs used for electrical stimulation, indicating how much current can be applied to each electrode.^39^ The CIL was tested by VTM upon applying a current-controlled sub millisecond stimulation pulse to the electrodes. Figure S3 shows the data for VTM of GMμEs and GMμEs/PEDOT:PSS and describes the experimental procedures for VTM. We found that the CIL improved from 0.27 ± 0.03 mC/cm^2^ (Pt) to 0.85 ± 0.08 mC/cm^2^ (PEDOT:PSS) and 3.54 ± 0.22 mC/cm^2^ (GMμ), respectively, and after combining to 4.96 ± 0.31 mC/cm^2^ (GMμ/PEDOT:PSS), Figure 3F, Table S1. By comparing the characteristics of CSC, CSC_C_, and CIL of the different electrodes, we found that the electrical stimulation performance of 3D-GMμEAs/PEDOT:PSS was excellent, suggesting that these electrodes were suitable for the electrical stimulation of neuronal networks.

### Stability of MEA

Achieving stable performance of electrodes is critical for regulating and monitoring neuronal networks. Here, we conducted a comprehensive characterization of the stability of our electrodes from two perspectives: electrical stimulation and long-term detection stability. To evaluate electrical stimulation stability, we applied intense stimulation and recorded impedance changes. We defined electrode failure (or delamination) as a 100% change in impedance at 1 kHz. Our results show that PEDOT:PSS-modified bare Pt electrodes delaminate quickly (∼2,000 pulses, Figure S4A). However, GMμEs and GMμEs/PEDOT:PSS exhibit greater stability, with GMμEs/PEDOT:PSS changing on average by less than 40% after 500,000 pulses, and GMμEs demonstrating even greater stability, with a change in impedance of less than 20% (on average) after 500,000 pulses, Figure S4B. These findings suggest that the stability is due to the GMμEs. Although PEDOT:PSS-modified bare Pt electrodes demonstrate instability after multiple pulse stimulation, their stability significantly improves when combined with GMμEs.

**Figure 4:**
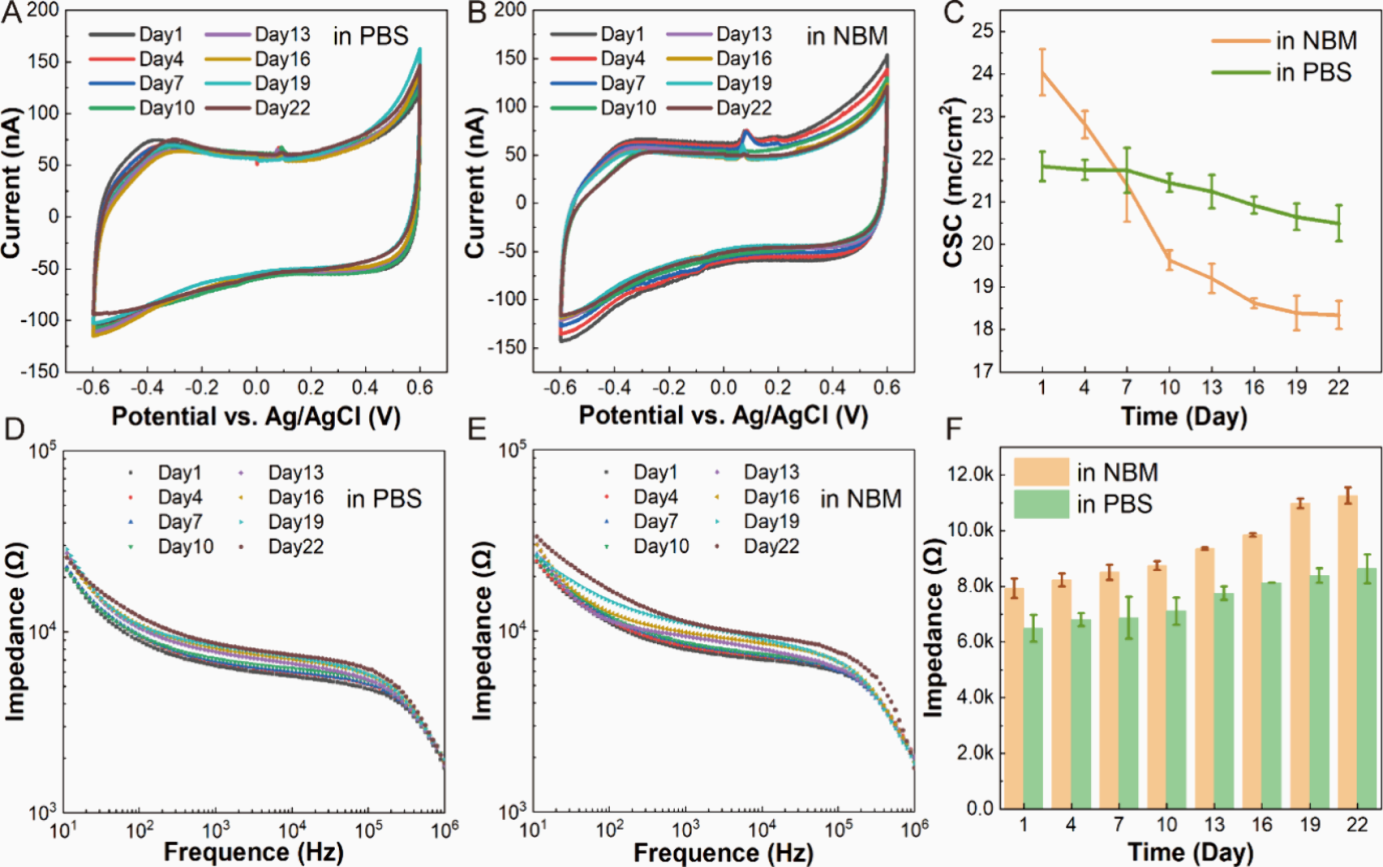
Stability characteristics of MEAs. (A) CV curves of GMμEs/PEDOT:PSS incubated in PBS. (B) CV curves of GMμEs/PEDOT:PSS incubated in NBM. (C) Time-dependent plots of CSC magnitude of GMμEs/PEDOT:PSS in PBS and NBM, showing a slight decrease over 22 days, *n* = 5. (D) Bode plots from EIS of GMμEs/PEDOT:PSS in PBS. (E) Bode plots from EIS of GMμEs/PEDOT:PSS in NBM. (F) Time-dependent plots of impedance magnitude of GMμEs/PEDOT:PSS at a frequency of 1 kHz, indicating a slight increase over time, *n* = 5.

To evaluate long-term detection stability, we characterized the MEAs by placing the electrodes in tissue culture media for more than 22 days and testing the CV and EIS curves through the period. CV curves, in Figure 4A and B, show that the oxidation peak of the 3D-GMμEAs/PEDOT:PSS in Neuro Basal Medium (NBM) and phosphate-buffered saline (PBS) remained almost unchanged from day 1 to day 22. The CSCs of the electrodes in NBM and PBS decreased slightly, and the statistical results showed that after 21 days, the CSCs were reduced by 23% in NBM and 6% in PBS, Figure 4C. The EIS curves of 3D-GMμEAs/PEDOT:PSS in NBM and PBS, Figure 4D and E, also showed little change in impedance up to 22 days. Unlike the CSC drop, the impedance of the electrode at 1kz rose slightly with time, Figure 4F, which may be due to two possible reasons. The first is the deposition of biomolecules, such as proteins, on the electrode surface over an extended period. The second is the hygroscopic swelling of PSS.^40,41^ These effects are unavoidable, so the electrodes in this study are stable and reliable for at least 3 weeks. However, to ensure that the electrode surfaces would outlast any electrical characterization measurements, we used them for culturing neurons and recorded pictures of neurons on electrodes for up to 4 weeks, Figure S5. These images showed that neurons formed a large number of synapses and network connections, indicating that the electrodes have excellent long-term stability and biocompatibility.

Considered together, we show that the 3D-GMμEAs/PEDOT:PSS have excellent electrical stimulation and long-term detection stability, enabling effective regulation of neurons by electrical stimulation, as well as promoting neuronal culture, synapse formation, and network connectivity. These findings have notable implications for developing stable electrodes for neuronal network regulation and monitoring applications.

### Detection Performance of MEA

The detection performance of 3D-GMμEAs/PEDOT:PSS electrodes was evaluated against planar electrodes by analyzing the spontaneous firing activity of neurons on six representative devices during the third week in the culture (Supporting Information S6). The electrophysiological signals of the cultured neurons on both types of devices have been successfully detected (Figure S6-1). However, the 3D-GMμEAs/PEDOT:PSS devices demonstrated a substantially higher number of active electrodes, a higher average firing rate of action potentials, as well as a higher amplitude and SNR of the detected neuronal signals, Figure S6-1, S6-2 and S6-3. These findings suggest that the 3D-GMμEAs/PEDOT:PSS devices significantly surpassed the planar electrode devices in all the metrics, showing superior performance in detection efficiency and sensitivity, which is highly desirable for neuronal network monitoring and regulation.

### Analysis of Spontaneous Activities of Dynamic Neuronal Networks

**Figure 5:**
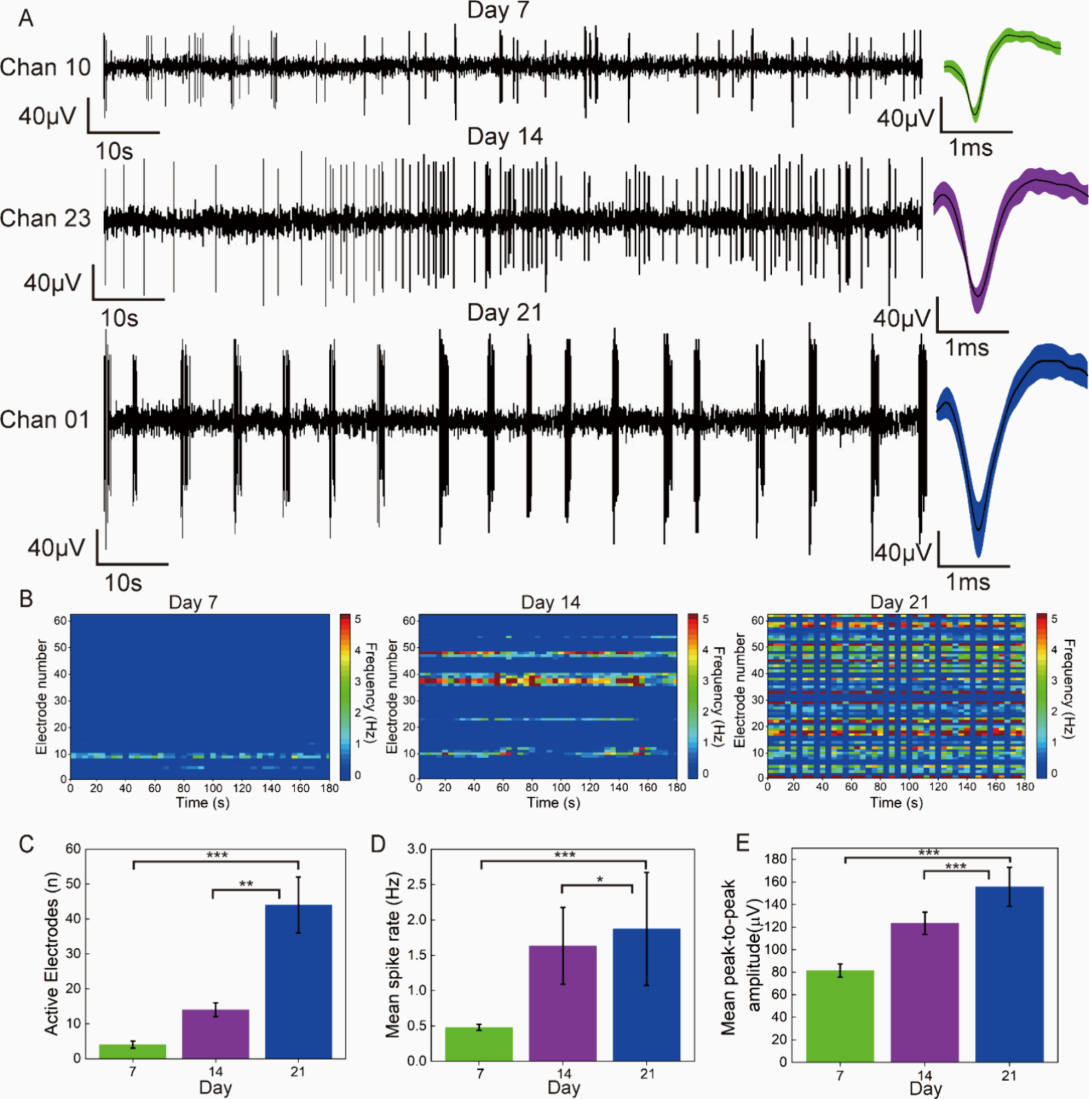
Temporal dynamics of spontaneous activities in 3D-GMμEA/PEDOT:PSS. (A) Representative spontaneous activities on different days *in vitro*: days 7, 14, and 21, including spike trains (left) and overlay spike patterns (right). (B) Color-mapped raster plots depicting spontaneous activities recorded from 62 electrodes of the 3D-GMμEA/PEDOT:PSS on days 7, 14, and 21. (C) Bar graph displaying the temporal evolution of the number of active electrodes on days 7, 14, and 21. (D) Bar graph showing the temporal evolution of the mean spike rate on days 7, 14, and 21. (E) Bar graph illustrating the temporal evolution of the mean peak-to-peak amplitude on days 7, 14, and 21. Note, for data in (C, D, E), *n* = 3 (three devices), **p < 0.01, ***p < 0.001***p < 0.001.

For an in-depth understanding of the network dynamics and the communication capability of functional connections, we conducted recordings and analyses of neuronal activity at different time points during *in vitro* culture, specifically on days 7, 14, and 21. As shown in Figure 5A, spontaneous activities emerged in certain electrodes from day 7 onward, with neuronal firing being sparse and the average spike amplitude being low. On day 14, the firing rate of neurons dramatically increased, and the average spike amplitude further increased. By day 21, the average spike amplitude continued to increase, and the neuronal firing exhibited a rhythmic pattern, which is indicative of neuronal maturation.^42−44^ The color-mapped raster plots of neurons in Figure 5B further illustrate the differences among neurons on different days. These statistical analyses demonstrate that the number of active electrodes, Figure 5C, mean spike rates, Figure 5D, and mean peak-to-peak amplitude of spikes, Figure 5E, all increased with culture time.

Furthermore, we recorded the number of burst firing of neurons at different time points during *in vitro* culture. Burst activity, which refers to the repetitive high-frequency firing of neurons, has been demonstrated to be crucial for interneuronal communication.^45,46^ Our findings showed that burst activity was detected in cultured neurons on day 7, became more prominent on day 14, and peaked on day 21 (Supporting Information S8, Table S2).

These analyses show that neurons cultured *in vitro* for 21 days reach a mature state, which is consistent with previous findings by other researchers.^47,48^ However, this information alone is insufficient to understand the state of the neuronal network. It is crucial to assess the communication capability of the network and information transmission between neurons in the corresponding state.

### Evaluation of Neuronal Networks Communication Capability

**Figure 6:**
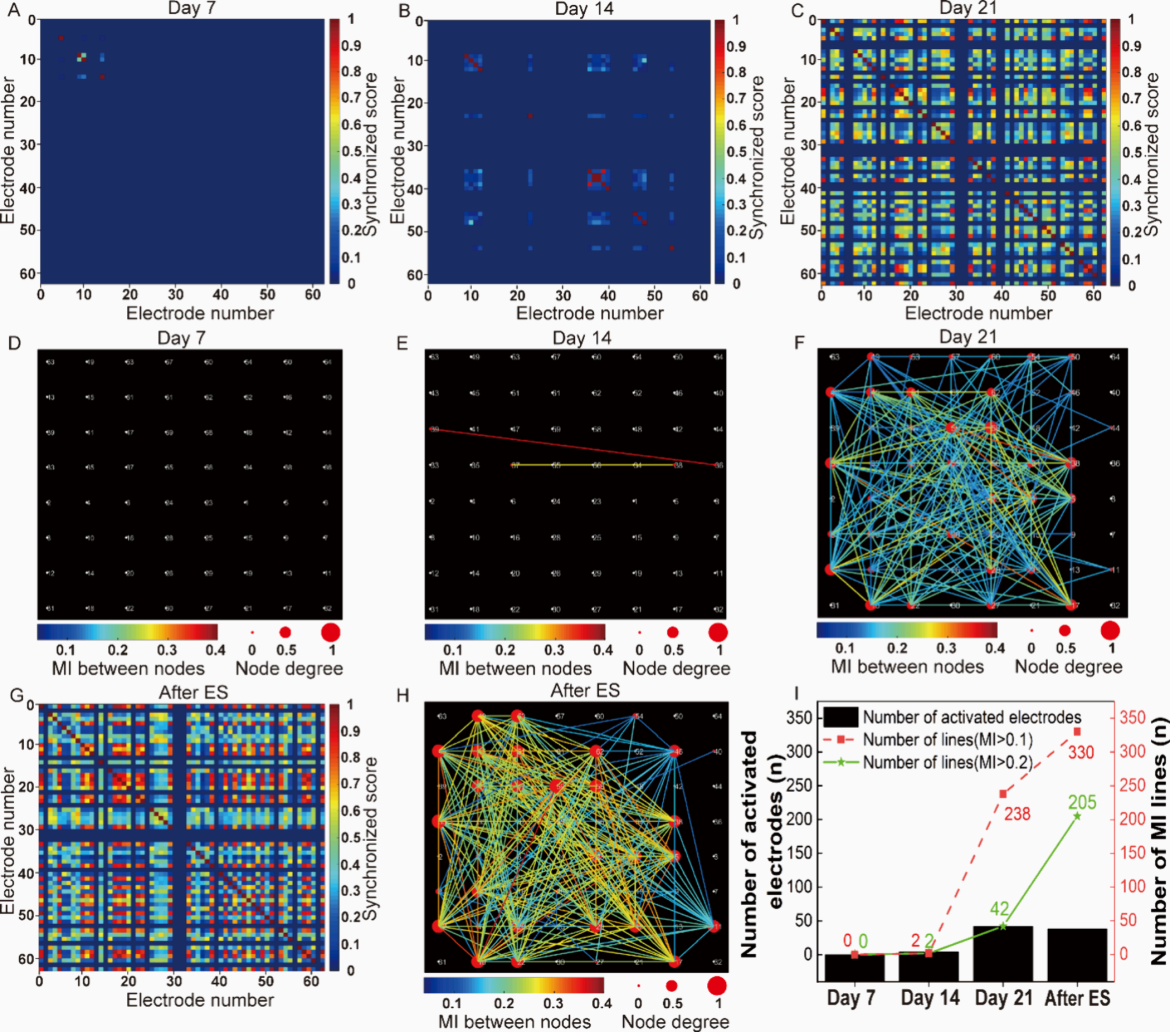
Evaluation of communication capability of dynamic neuronal networks. (A–C) Color-mapped cross-correlation matrices illustrating synchronized scores between electrodes based on spontaneous activities at different days *in vitro*: day 7 (A), day 14 (B), and day 21 (C). (D–F) Network maps depicting connectivity with node degrees, as well as the mutual information (MI) between nodes based on spontaneous activities on different days *in vitro*: day 7 (D), day 14 (E), and day 21 (F). The color of the lines indicated the MI between nodes. Larger nodes represent more connected nodes. (G–H) Color-mapped cross-correlation matrix and network map showing changes following electrical stimulation (ES) at day 21. (I) Bar graphs showing the number of activated electrodes, the number of connected lines, and the number of connected lines (MI > 0.2) in the network based on spontaneous activities on day 7, 14, and 21 and after electrical stimulation for day 21.

The concept and characterization method of neuronal network communication capability was elaborated in Supporting Information S7. Based on this concept, we first employed a synchronization approach to calculate the correlation between pairs of neurons, assigning a score that ranged from zero (lowest) to one (highest) to each pair of electrodes. A higher score indicated a greater level of synchronization between neurons, which suggested that they may belong to the same functional neuronal network.^49,50^ It was found that synchronization between active electrodes increased with culture time and the number of active electrodes. Specifically, we observed a notable increase in the number and synchronization of active electrodes on day 21 compared to day 14 and day 7, Figure 6A-C, Color-mapped cross-correlation matrices displaying synchronized scores between electrodes. These results demonstrate that, compared to the networks of less mature neurons (day 14 and day 7), networks of mature neurons (day 21) feature a greater number of interconnections among neurons.

Using JIDT,^51^ an information-theoretic toolkit for studying complex system dynamics, we calculated the mutual information (MI) between neurons. We then visualized the communication network between neurons using a custom program. Each electrode was represented as a node, with MI between electrodes serving as the weight. We considered a connection between two nodes as a communication network if the MI between them was >0.1 and introduced a node degree to distinguish the number of connections. It was found that day 7 lacked any network connections, Figure 6D, while day 14 had only a few connections with nodes exhibiting a relatively high MI, Figure 6E. In contrast, day 21 was characterized by a significantly larger number of network connections, as evidenced by the 42 activated electrodes and 238 connected lines, with 42 lines having an MI > 0.2, which is much higher than the previous 2 weeks, Figure 6I. These findings demonstrate an obvious increase in communication connectivity between neurons with increasing culture time. Furthermore, based on our comparative analysis of network communication capability, it is evident that mature neuronal networks exhibit superior overall communication capability compared to their immature counterparts (Figure S7).

Although we assessed the communication capability within the networks, we cannot ascertain whether these communication connections are based on actual mature synaptic connections. The hallmark of mature synaptic connections is the plasticity of neuronal networks. Since electrical stimulation has been shown to be effective in both regulating and evaluating neuronal network plasticity,^52,53^ we electrically stimulated the neuronal network formed at day 21 to measure this. After stimulation, we observed a notable increase in the overall degree of synchronization compared to before stimulation, Figure 6C, G. The MI-based neuronal network also displayed notable differences before and after stimulation, Figure 6F, H. Specifically, the number of connecting lines between nodes reached 330 after stimulation, which was 92 more than before stimulation. The MI scores between electrodes were greater after stimulation, with the number of lines with MI > 0.2 increasing by 163, Figure 6I. Notably, the increase in MI > 0.2 after electrical stimulation was greater than that in MI > 0.1, indicating that electrical stimulation primarily increased pre-existing communication connectivity. These results demonstrate that the communication capability is based on actual neuronal synaptic communication, and the electrical stimulation can enhance the neuronal network communication capability (Figure S7).

### Communication Connectivity for Neuronal Networks

**Figure 7:**
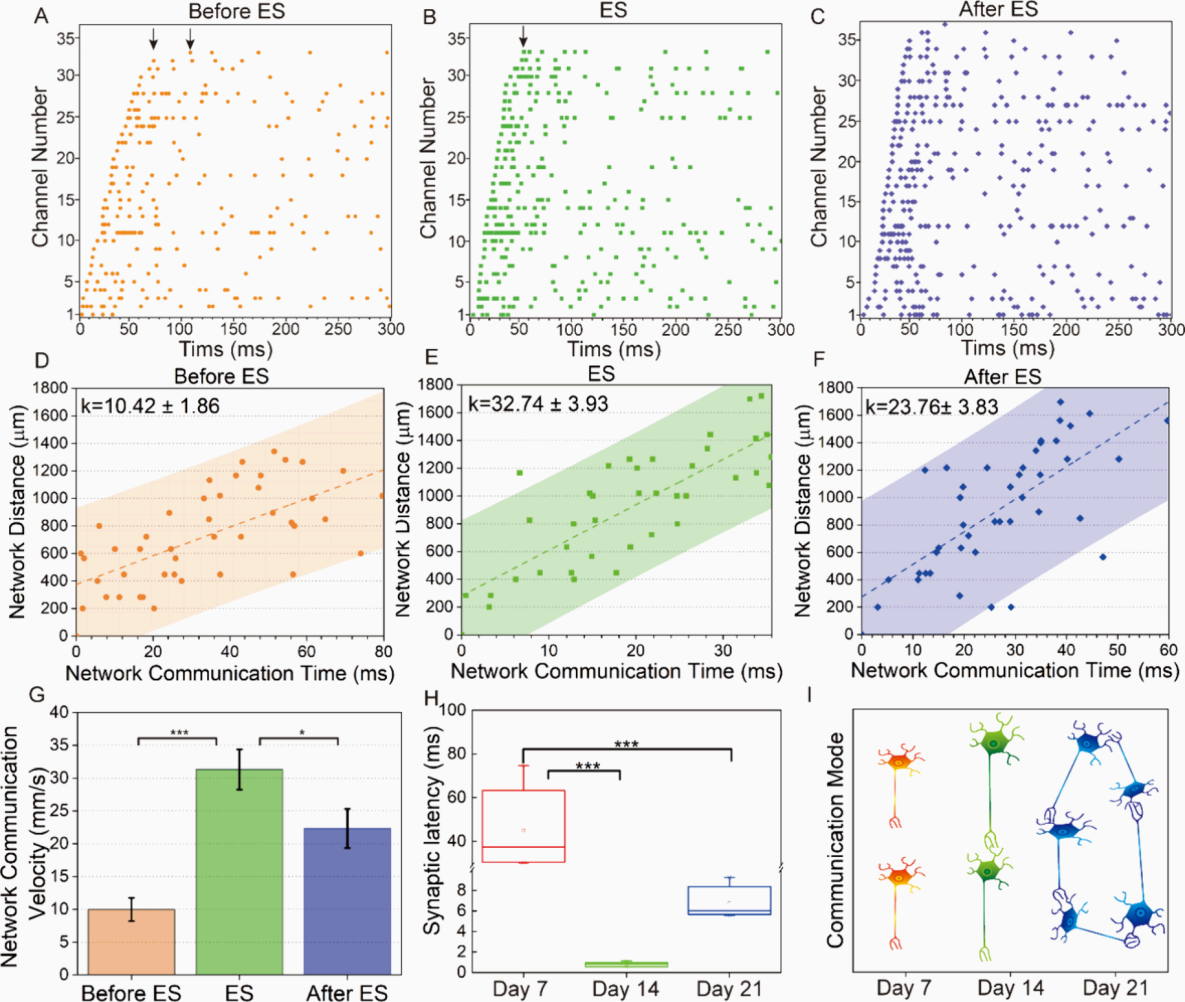
Synaptic latency (SL) and network communication speed. (A–C) Time series of partial spikes in a burst of neurons before, during, and after electrical stimulation (ES). (D–F) Scatter plot of network distance to all firing sites from the first firing site depending on network communication time before, during, and after electrical stimulation. The dotted line and shaded area represent the best fit of linear regression and the 95% confidence level, respectively. The slope of the linear regression denotes the network communication velocity. (G) Bar graph displaying the network communication velocity across the before, during, and after electrical stimulation states, *n* = 5, *p < 0.05, *** *p* < 0.001. Note, data distribution is included in Figure S10-2. (H) Box-plots illustrating changes in synaptic latency (SL) across days *in vitro*, *n* = 3, *** *p* < 0.001. Note, data distribution is included in Figure S11-2. (I) Plot depicting the communication connectivity mode across days *in vitro*.

Having established a macroscopical approach to evaluate the communication capability of the neuronal network, it remains unclear what factors contribute to communication variations at different developmental stages of neuronal networks. Thus, we further investigated the neuronal networks from a microscopic perspective by analyzing the synaptic transmission process locally at different states. It was found that there is a delay in information transfer between two neurons, referred to as synaptic latency (SL), which provides direct evidence of communication connectivity between neurons.^54^ For the sake of simplicity, we define the communication time in a neuronal network as the time difference from the start of the first neuron delivery to the last neuron delivery when the difference between adjacent delivery times is not greater than the maximum SL time (10 ms), see Supplementary S9. Based on our algorithm, we sorted the network communication time before electrical stimulation, during electrical stimulation, and after electrical stimulation. Figure 7 A-C shows the discharge time series of action potentials in different stages of the neuronal network within a burst. We found that the network communication time differed in the three states mentioned above; specifically, during stimulation, Figure 7B, was shorter than after stimulation, Figure 7C, which was shorter than before stimulation, Figure 7A. This phenomenon also appeared for the total time of the entire burst, Figure S9. This may be due to the regulation of the SL time of neurons by electrical stimulation, which further changes the communication duration of the neuronal network.

To confirm our hypothesis, we introduce the network communication speed to quantify this process, see Figure S10–1 and Supporting Information S10. As shown in Figure 7D-F, the linear regression slope of 2D data points of network communication time versus the network distance represents the network communication speed. Statistical analysis shows that the communication speed during stimulation was faster than after and before stimulation, Figure 7G and Figure S10-2. These results indicate that electrical stimulation can change the communication speed of the network by changing the SL between neurons. Faster information transfer between neurons can lead to higher overall correlation and larger MI in the neuronal network.

Considering that the neuronal networks on day 7 and day 14 have not yet reached maturity, we further conducted a statistical analysis of SL between neurons on different days, Figure S11-1, Figure S11-2, and Figure 7H. The SL on day 7 was much greater than 10 ms, indicating that there were no synaptic connections between neurons and neurons were discharging randomly. The SL on day 14 was the smallest, less than 1 ms, almost the smallest time reported in the literature, indicating that the neurons were directly connected without intermediate neurons acting as relay transmitters.^55^ The neurons cultivated on day 21 had many synapses between multiple electrode points, so the SL between neurons at the same distance as day 14 was approximately 6 ms, indicating that an increase in synapses slowed down the speed of information transfer between neurons.

In summary, the connecting pattern between neurons constantly changes as their network develops and determines the network communication connectivity. The communication connectivity model can be described in Supporting Information S12 and the following Figure 7I: on day 7, neurons have not yet formed connections and discharge randomly without a communication connectivity network (also see Figure 6D); on day 14, neurons are directly connected with the smallest SL, forming a large MI communication connectivity (also see Figure 6E); on day 21, a large number of neurons are connected to form a mature neuronal network and information transfer needs to go through multiple synapses (also see Figure 6F).

## Conclusion

Sensing neural signal at electrode arrays whose elements have high SNR, good stability, and whose fabrication and functionalization can be precisely controlled have long been in demand for studying neuronal network dynamics, *in vitro*. To meet these requirements, we developed a customizable 3D gold microelectrode array modified with PEDOT:PSS that can be used for neuron culture with both spatial and temporal control of neural information recording, and local electrical stimulation to regulate neurons. We show that such fabricated devices possess excellent electrical properties, including low impedance, low phase delay, high CSC and CSC_C_ and high CIL. Importantly, the electrode array also exhibited outstanding electrical stimulation stability, long-term detection stability, and biocompatibility enabling extended studies to be performed, successfully detecting neurons’ discharge information *in vitro* for 3 weeks and capturing synaptic latencies between neurons. Such capability is essential for studying communication within neuronal networks both locally (i.e., individual neurons) and macroscopically (i.e., the whole network), obtaining rich and invaluable information for an in-depth understanding of communication dynamics at different development stages of neuronal networks.

We demonstrated that correlation heat maps and mutual information networks can effectively evaluate the communication capability within the neuronal networks. With the outstanding performance of the device, we were able to conduct an actual neuronal network verification through electrical stimulation, which validated the nature of synaptic connections for network communication.

Importantly, through in-depth analysis of communication network variations, we have developed a communication connectivity model for neuronal network development stages. We show that, for an immature neuronal network, the connection mode between neurons determines the communication capability. As the network progresses toward maturity, the connection mode changes and the communication capability was enhanced. Finally, for a mature neuronal network, electrical stimulation speeds up the synaptic latency between neurons, altering the communication speed and ultimately changing communication patterns in the network.

It is worth noting that our electrode fabrication approach can be applied to flexible substrates and in future could be utilized for long-term *in vivo* monitoring. The devices could be readily integrated with other accessories, such as microfluidics, for real-time and *in situ* investigations of neuronal networks. In the future, as the demand for cell detection continues to grow, technologies like these will have greater potential for application.^56^ The method for evaluating communication capability and the communication connectivity model can be used for not only neuronal networks but neural networks in general, which can be applied in a wide range of applications, from the fundamental investigations of neurological disorders to developing devices and materials for therapies.

## Methods

### Reagents and Apparatus

Poly(sodium-4-styrenesulfonate) (PSS) was acquired from HEROCHEM, China, while ethylene deoxy thiophene (EDOT) was purchased from Aladdin, China. The 24k pure gold plating solution was obtained from Tianyue (China), and phosphate-buffered saline (PBS, 0.1 M, pH 7.4) and glutamate sodium (Glu, ≥99%) were procured from Shanghai Chemical Reagent Company. HBSS buffer, DNase, papain, DMEM buffer, and Neurobasal Plus Medium were acquired from Sigma-Aldrich, while cytarabine was purchased from Thermo Fisher.

Interface modification of the MEAs was conducted using the electrochemical workstation (Gamry Reference 600, Gamry Instruments). Electrophysiological signals were recorded using a 128-channel neural data recording system (Blackrock Microsystems). Other equipment, including scanning electron microscope, preamplifier (Blackrock Microsystems), CO_2_ incubator (Thermo Fisher), the dual-channel electrophysiological electrical stimulator (Multichannel), and oscilloscope (TPS2024, Tektronix), were also utilized in the experimental procedures.

### Fabrication of the 3D-GmμEAs/PEDOT:PSS

The electrodes were fabricated on a 5 cm × 5 cm quartz glass substrate using standard microelectromechanical system technologies and electroplating techniques. The flowchart of the electrode fabrication process is presented in Figure S13A-K, which can be divided into four parts: 1) Patterning the electrodes on the substrate by utilizing the first layer of the photolithographic mask plate (Figure S13A-D). 2) Formation of a three-dimensional “mushroom”-like gold structure on top of the planar electrodes via electroplating with the protection of photoresist AZ4903 (Figure S13E-G). 3) Deposition of an insulating layer, followed by selective etching to expose the GMμEs, reference electrodes, and external pads (Figure S13H-J). 4) Surface modification of the GMμEs with PEDOT:PSS (Figure S13K).

### Process of Electroplating GMμEs

Upon completion of the process steps depicted in Figure S13D, the samples underwent thorough cleaning with acetone, isopropanol, and water, and were subsequently dried at 80 °C for 5 min. Additional cleaning with O_2_ plasma (100W, 30s) was performed to enhance hydrophilicity of the electrode surface and improve adhesion to the photoresist. As shown in Figure S13E, the positive photoresist AZ4903 was uniformly spin-coated onto the planar electrode at a shaking speed of 1500 r/min for 1 min, followed by gradient baking on a hot plate. Finally, the photoresist was exposed by a mask plate with the same pattern as the planar electrode sites. As shown in Figure S13F, the exposed electrode was shaken evenly and slowly in the developer for about 5 min to remove the exposed area, followed by cleaning with water and removing the photoresist residue through plasma cleaning (100W, 1 min).

To initiate gold plating, the conductive tape was used to short-circuit the external pads of the electrodes in the array, Figure S14B, and connected to a common wire to make them into the working electrode during gold electrodeposition, Figure S14C. A 24k gold plating solution (with sodium gold sulfite as the main salt component, Figure S13M), was used to create the 3D gold structure, along with an external platinum reference electrode, Figure S14A. The plating was performed using a two-electrode system with a constant plating voltage, applied through an electrochemical workstation with monitoring of the current density throughout. The electroplating parameters are typically set at a voltage of −1.5 V and a duration of approximately 1 h. Overfilling of the micropores was necessary to grow “mushroom”-like gold structures during the electrodeposition step; otherwise, columns without a cap would be created. The height of the 3D gold structure could be customized by varying the thickness of the photoresist and the time of electroplating. The effect of electroplating parameters on electrode height and electroplating stability was demonstrated in Supporting Information S15.

### Process of Electroplating PEDOT:PSS

After the fabrication of the GMμE, a further modification was necessary to improve its performance. The chosen modification was the deposition of the conductive polymer PEDOT:PSS onto the surface of the GMμEs, as illustrated in Figures S13K and Supplementary S13M. Prior to the modification, the electrode surface was meticulously cleaned with O_2_ plasma (100 W, 3 min). Subsequently, the PEDOT:PSS was electrodeposited onto the electrode using a three-electrode system, Figure S16. The working electrode was the GMμE site to be modified, the counter electrode was a Pt electrode, and the reference electrode was Ag/AgCl. The plating solution was obtained by mixing 0.02 M EDOT and 0.1 M PSS and sonicating for 30 min. The PEDOT:PSS deposition was performed by CV with a potential range of 0 to 0.95 V and a scan rate of 50 mV/s for 12 cycles.

### Primary Hippocampal Neuron Culture

All animal surgeries, including anesthesia and euthanasia, complied with the ethical guidelines of the Chinese Academy of Medical Sciences- Peking Union Medical College. The research team involved in animal experimentation held valid Beijing Laboratory Animal Practitioner Qualification Certificates. For the isolation and culture of primary hippocampal neurons, pregnant Chronotropic Cancer Institute mice were euthanized on embryonic day 15.5. The uterus was removed and placed in prechilled HBSS buffer, followed by careful dissection of the fetus and separation of the hippocampus. After removing the meninges, the tissue was minced and treated with DNase and papain in DMEM buffer for 15 min. Gentle pipetting was employed to dissociate the tissue, and the supernatant was collected after centrifugation at 100 g for 5 min. The cells were then resuspended in Neurobasal Plus culture medium, counted using a hemocytometer, and seeded onto MEAs at a concentration of 1 × 10^6^ cells/mL. The devices with cells were maintained in a humidified CO_2_ incubator at 37 °C for cell culture, and 10 μM asiatic cytidine was added on the second day to prevent excessive glial cell growth. The culture medium was replaced twice a week by replacing 50% of the existing medium until further use.

### Neuronal Recording and Electrical Stimulation

Electrophysiological signals of neurons were recorded using electrodes and amplified by a custom electrode interface, Figure S17, at a sampling rate of 20 kHz per channel. The high-pass filter (>250 Hz) and low-pass filter (<250 Hz) were used to acquire neuronal action potentials (Spike) and local field potentials (LFP), respectively. Data were monitored and recorded with MC Rack software (Blackrock Microsystems). Mature cultured neurons were subjected to electrical stimulation using a dual-channel electrophysiological electrical stimulator. A negative-phase-leading biphasic rectangular voltage pulse with an amplitude of 300 mV, pulse length of 200 μs, and frequency of 1 Hz was used for training. During stimulation, the ground wire of the electrical stimulator was connected to the ground of the MEA interface circuit, and the stimulating electrodes of the electrical stimulator were selectively connected to arbitrary active electrode sites. The stimulating electrode was disconnected during recording to avoid any artifacts in the recorded signal.

### Electrophysiological Analysis

The recorded spikes and LFPs data were analyzed using the Offline Sorter and Neuroexplorer software programs. To extract neural spikes, the principal component analysis (PCA) method was used to isolate the first three principal components, and the Valley-Seeking algorithm was then used to sort out single-unit spikes. Every single-unit spike was regarded as an action potential train from a neuron. The sorted data and LFP signals were further analyzed. All statistical analyses were performed in MATLAB (Math Works), Python (Python Software Foundation), JIDT (Java Information Dynamics Toolkit) or Origin 2018 (Origin Lab). The data were presented as mean ± standard error of the mean. At least 3 devices were used for each condition. A two-tailed *t* test was used to compare the mean values for the two groups. A significance level of *P* < 0.05 was established for all analyses.

## Supporting Information Available

Texts S1–S17, Figures S1–S17, and Tables S1 and S2. S1: Morphological characteristics of the MEA. Figure S1: More examples of the morphological characterization of the MEA. S2: Electrical characteristics of MEA. Figure S2: Measurements of the C_dl_ from CV experiments. S3: Electrical stimulation of MEA. Figure S3: Voltage transient detection of GMμEs and GMμEs/PEDOT:PSS. Table S1: Electrical and electrical stimulation properties characteristics of MEA. S4: Stability characteristics of MEA. Figure S4: Electrical stimulation stability of PEDOT:PSS-modified bare Pt electrodes, GMμEs, and GMμEs/PEDOT:PSS. S5: Biocompatibility of 3D-GMμEAs/PEDOT:PSS. Figure S5: Pictures of neurons on electrodes for up to 4 weeks. S6: Detection performance of MEA. Figure S6-1: Comparison of active channels between 3D-GMμEAs/PEDOT:PSS and planar electrodes. Figure S6-2: Raw data recordings from randomly selected nine channels (i.e., active electrodes) of a representative device of each type. Figure S6-3: Comparison of detection performance between planar and 3D-GMμEAs/PEDOT:PSS electrodes. S7: Characterization of neuronal network communication capability. Figure S7: Assessment of neuronal network communication capability at different stages. S8: Analysis of spontaneous activity of neurons across days. Table S2: Statistics of bursts of neurons across days. S9: Calculation of network communication time. Figure S9: Time series of entire spikes in a burst of neurons. S10: Calculation of network communication speed. Figure S10-1: Calculation of network communication speed. Figure S10-2: Scatter plot of network distance to all firing sites from the first firing site depending on network communication time before, during, and after electrical stimulation. S11: Calculation of synaptic delay across days. Figure S11-1: Synaptic delay across days in vitro. Figure S11-2: Data distribution of synaptic delay times for Figure 7H. S12: Explanation of the neuronal network communication connectivity model. S13: Fabrication of the 3D-GMμEAs/PEDOT:PSS. Figure S13: Fabrication and modification of 3D-GMμEAs/PEDOT:PSS. S14: Fabrication of GMμEs. Figure S14: Electroplating methods for GMμEs. S15: Effect of electroplating parameters on electrode height and electroplating stability. Figure S15-1: Schematic illustration of electrode structural changes during the electroplating process. Figure S15-2: Effect of electroplating parameters on electroplating height and electroplating stability. Figure S15-3: Characterization of electroplating parameter stability. S16: Process of electroplating PEDOT:PSS. Figure S16: Three-electrode system electroplating PEDOT:PSS. S17: Neuronal recording and electrical stimulation. Figure S17: Circuit interface of MEA. (PDF)
