# Supplementary material for: Investigating Communication Dynamics in Neuronal Network using 3D Gold Microelectrode Arrays
Source: ACS Nano. 2024 Jun 20;18(26):17162–74. doi: 10.1021/acsnano.4c03983 (PMC11349149; doi:10.1021/acsnano.4c03983)
Supplement: Supplementary file 1 — nn4c03983_si_001.pdf [file nn4c03983_si_001.pdf]

Supporting information

for

Investigating Communication Dynamics in Neuronal Network using 3D Gold Microelectrode Arrays

Kui Zhang[1 2], Yu Deng[3], Yaoyao Liu[1 2], Jinping Luo[1 2], Andrew Glidle[4], Jonathan M. Cooper [4], Shihong Xu[1 2], Yan Yang[1 2], Shiya Lv[1 2], Zhaojie Xu[1 2], Yirong Wu[1 2], Longzhe Sha[3], Qi Xu[3], Huabing Yin [4*], and Xinxia Cai[1 2 *].*

[1] State Key Laboratory of Transducer Technology, Aerospace Information Research Institute, Chinese Academy of Sciences, Beijing 100190, China;

[2] School of Electronic, Electrical and Communication Engineering, University of Chinese Academy of Sciences, Beijing 100049, China;

[3] State Key Laboratory of Medical Molecular Biology, Institute of Basic Medical Sciences, Chinese Academy of Medical Sciences and Peking Union Medical College, Beijing 100005, China;

[4] James Watt School of Engineering, University of Glasgow, G12 8LT, UK

*Corresponding Author: **xuqi@pumc.edu.cn (Q.X.); Huabing.Yin@glasgow.ac.uk (H.Y.); xxcai@mail.ie.ac.cn (X.C.)**

S1. Morphological characteristics of the MEA.

Figure S1. More examples of the morphological characterization of the MEA.

S2. Electrical characteristics of MEA.

Figure S2. Measurements of the C_{dl} from CV experiments.

S3. Electrical stimulation of MEA.

Figure S3. The voltage transient detection of GMμEs and GMμEs/PEDOT:PSS.

Table S1. Electrical and electrical stimulation properties characteristics of MEA.

S4. Stability characteristics of MEA.

Figure S4. Electrical stimulation stability of PEDOT:PSS-modified bare Pt electrodes, GMμEs and GMμEs/PEDOT:PSS.

S5. Biocompatibility of 3D-GMμEAs/PEDOT:PSS.

Figure S5. Pictures of neurons on electrodes for up to four weeks.

S6. Detection performance of MEA.

Figure S6-1. Comparison of active channels between 3D-GMμEAs/PEDOT:PSS and planar electrodes.

Figure S6-2. Raw data recordings from randomly selected nine channels (*i.e.* active electrodes) of a representative device of each type.

Figure S6-3. Comparison of detection performance between planar and 3D-GMμEAs/PEDOT:PSS electrodes.

S7. Characterization of neuronal network communication capability.

Figure S7. Assessment of neuronal network communication capability at different stages.

S8. Analysis of spontaneous activity of neurons across days

Table S2. Statistics of bursts of neurons across days

S9. Calculation of network communication time.

Figure S9. Time series of entire spikes in a burst of neurons.

S10. Calculation of network communication speed.

Figure S10-1. Calculation of network communication speed.

Figure S10-2. Scatter plot of network distance to all firing sites from the first firing site depending on network communication time before, during, and after electrical stimulation.

S11. Calculation of synaptic delay across days.

Figure S11-1. Synaptic delay across days in vitro.

Figure S11-2. Data distribution of synaptic delay times for Figure 7H.

S12. Explanation of the neuronal network communication connectivity model

S13. Fabrication of the 3D-GMμEAs/PEDOT:PSS.

Figure S13. Fabrication and modification of the 3D-GMμEAs/PEDOT:PSS.

S14. Fabrication of GMμEs.

Figure S14. Electroplating methods for GMμEs.

S15. The effect of electroplating parameters on electrode height and electroplating stability.

Figure S15-1. Schematic illustration of electrode structural changes during the electroplating process.

Figure S15-2. The effect of electroplating parameters on electroplating height and electroplating stability.

Figure S15-3. Characterization of electroplating parameter stability.

S16. Process of electroplating PEDOT:PSS.

Figure S16. Three-electrode system electroplating PEDOT:PSS.

S17. Neuronal recording and electrical stimulation.

Figure S17. The circuit interface of MEA.

S1 Morphological characteristics of the MEA.

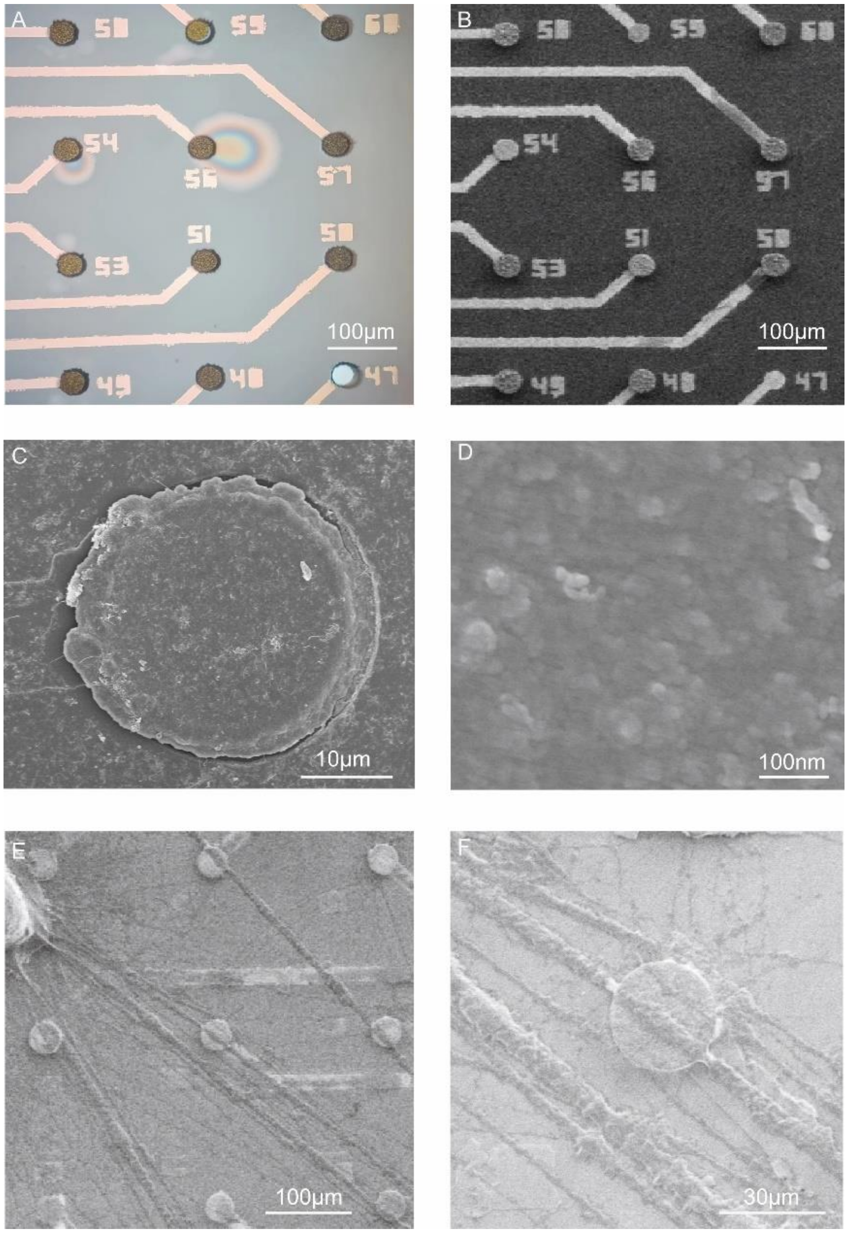

Figure S1. More examples of the morphological characterization of the MEA. (A, B) Micrograph and SEM image of multiple electrode sites under selective plating. (C) SEM image of a single PEDOT:PSS-modified bare Pt electrode. (D) SEM image of GMμE/ PEDOT:PSS surface. (E, F) SEM images of neurons cultured on electrodes.

Supplementary Material S14 details a selective plating method that allows for modifying individual or groups of electrodes. Specifically, Site No. 47 in Supplementary Figure S1 A,B displays the bare electrode interface resulting from selective plating without plating, thereby enabling electrodes of varying heights within the same MEA for customized research. Supplementary Figure S1 C, D present the morphology of PEDOT:PSS. Panel S1C shows the CV-based PEDOT:PSS modification on the bare electrode, referred to as the planar electrode in this study, for comparison with the detection performance of 3D-GMμEAs/PEDOT:PSS. In panel S1D,

the nanostructure of PEDOT:PSS modified on the golden electrode demonstrates numerous rough structures on the surface, thereby significantly increasing the electrode's specific surface area and enhancing its performance. Supplementary Figure S1 E, F presents an SEM image of neurons cultured for four weeks on electrodes, illustrating the spatial relationship between neurons and electrodes.

S2 Electrical characteristics of MEA.

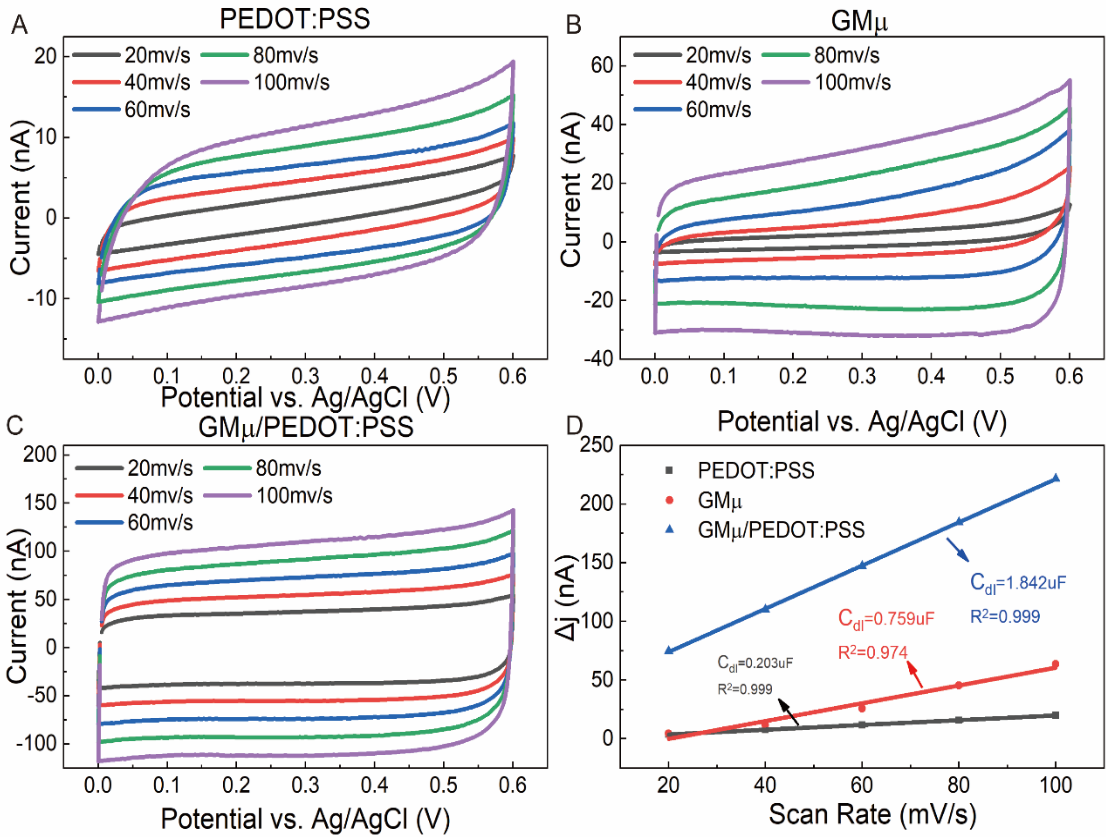

Figure S2. Measurements of the C_{dl} from CV experiments. (A, B, C) The CV curves of PEDOT:PSS-modified bare Pt electrodes (A), GMμEs (B), and GMμEs/PEDOT:PSS (C) at different scan rates. (D) The double layer capacitance of the GMμEs/PEDOT:PSS, GMμEs and PEDOT:PSS-modified bare Pt electrodes.

The electric double-layer capacitance (C_{dl}) is a crucial parameter that characterizes the activity of the electrode surface, which refers to the capacitance formed between the electrode surface and the electrolyte. One commonly used method for calculating this capacitance is CV, which can provide information on the change of electrode surface potential with time, and the corresponding change in current with time. The relationship between the current and scan rate of the forward and reverse scans in the CV curve can be used to calculate the electrode surface capacitance. Specifically, the slope resulting from dividing the difference between the current values of the forward and reverse scans by the scan rate represents the surface capacitance of the electrode. Since the influence of other capacitances is minimal in this experiment, the calculated value of the surface capacitance is used as an approximation for the electric double-layer capacitance.

The results indicate that GMμEs/PEDOT:PSS exhibited a significantly higher C_{dl} value (1.84 μF), indicating a larger active surface area and more active sites compared to those of GMμEs (C_{dl} = 0.76 μF) and PEDOT:PSS-modified bare Pt electrodes (C_{dl} = 0.20 μF). Based on the above, it can be concluded that the GMμEs/PEDOT:PSS modification method is more effective in enhancing the electrode surface activity and improving the performance of electrodes.

S3 Electrical stimulation of MEA.

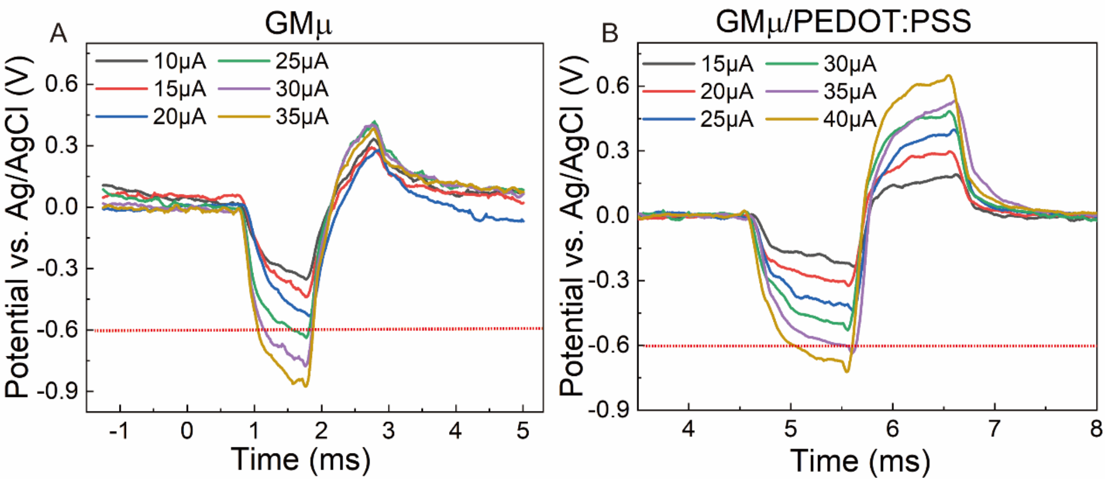

Figure S3. **The voltage transient detection of GMμEs and GMμEs/PEDOT: PSS.** (A) GMμEs (B) GMμEs/PEDOT:PSS.

The charge injection limit (CIL) is measured in PBS solution by the electric potential transient method. The measurement also adopts a three-electrode system, and the counter electrode and reference electrode are Pt electrode and Ag/AgCl electrode, respectively. During the measurement, a dual-channel electrophysiological electrical stimulator is used to apply a current electrical stimulation pulse between the working electrode and the counter electrode, and at the same time an oscilloscope is used to record between the working electrode and the reference electrode to measure the corresponding curve of its potential, Figure S3. By continuously increasing the current value of the stimulation pulse, the potential response under different current pulses is recorded. When the potential polarization curve exceeds the potential window (Figure S3), the electrical stimulation current value is the maximum safe current. The injected charge density is the maximum safe charge injection amount. The CIL calculation method is as follows:

$$CIL = (Imax \times t)/S$$

Where *Imax* is the maximum injection current, *t* is the pulse width, and *S* is the electrode area. As depicted in Figure S3AB, the electrodes exhibit Imax of 25 μA (GMμ) and 35 μA (GMμ/PEDOT:PSS), respectively. Based on calculations, the ***CIL*** is 3.54±0.22 mC/cm^2 (GMμ) and 4.96±0.31 mC/cm^2 (GMμ/PEDOT:PSS). Taken together, 3D-GMμEAs/PEDOT:PSS demonstrates superior capacitive performance, enabling the storage of more charges at a given potential.

Table S1 Electrical and electrical stimulation properties characteristics of MEA.

Characteristics / Material	Impedance (kΩ) at 1 kHz	Phase (°) at 1 kHz	CSC (mC/cm^2)	CSC$_C$ (mC/cm^2)	CIL (mC/cm^2)
Pt	341.02 ± 22.40	-68.56 ± 1.21	0.35 ± 0.03	0.16 ± 0.04	0.27 ± 0.03
PEDOT:PSS	29.98 ± 8.34	-20.52 ± 1.71	2.55 ± 0.31	1.18 ± 0.15	0.85 ± 0.08
GMμ	26.51 ± 3.24	-57.53 ± 3.24	8.05 ± 0.23	5.05 ± 0.04	3.54 ± 0.22
GMμ/PEDOT:PSS	6.49 ± 0.67	-16.40 ± 0.74	24.11 ± 0.46	11.26 ± 0.52	4.96 ± 0.31

S4 Stability characteristics of MEA

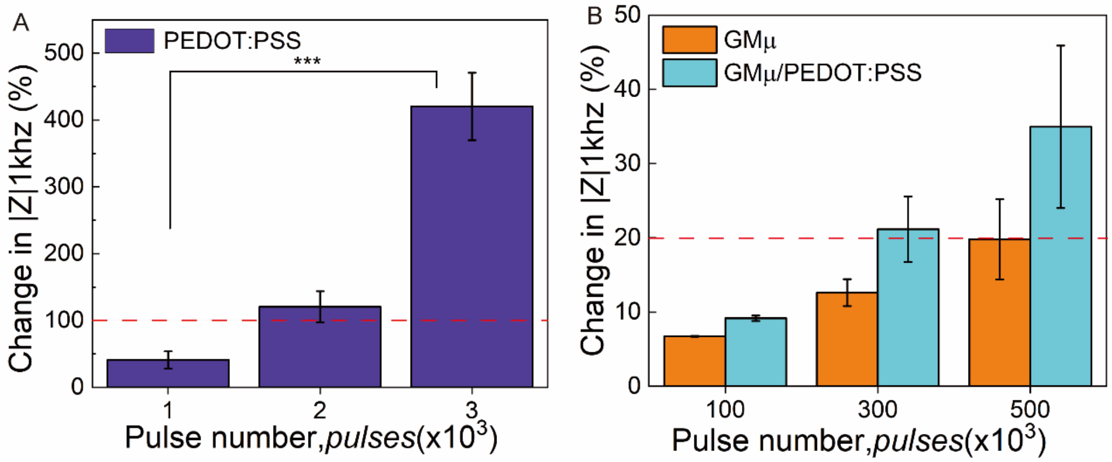

Figure S4. Electrical stimulation stability of (A) PEDOT:PSS-modified bare Pt electrodes, n=5, *** p <0.001, and (B) GMμEs and GMμEs/PEDOT:PSS, n=5.

S5 Biocompatibility characterization of MEA

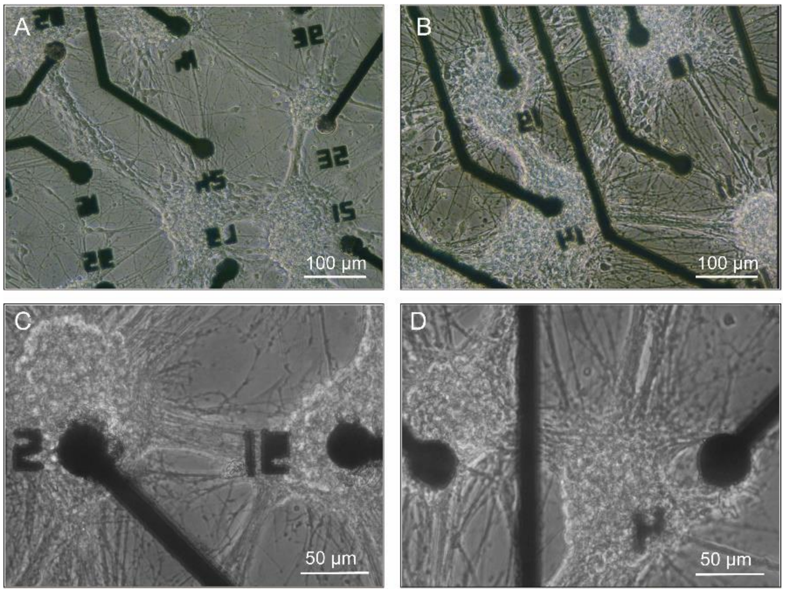

Figure S5. Pictures of neurons on electrodes for up to four weeks.

By observing images of neurons cultured on the electrode surface for four weeks, we can see that the synapses of neurons have formed a good connection with the electrode surface, indicating a close interaction between the material on the electrode surface and neurons. This connection also indicates that the electrode has good biocompatibility with neurons, meaning it will not cause significant damage to neurons. Furthermore, neurons extend substantially on the electrode surface, which is another evidence of the electrode's good biocompatibility.

S6 Detection performance of MEA

To characterize the detection performance of the 3D-GMμEAs/PEDOT:PSS electrodes, we performed neuronal recording testing on randomly selected 3D-GMμEAs/PEDOT:PSS devices and planar devices (three devices per type) independently. Neurons were seeded at $1x10^6$ cells/cm^2 for all the experiments, and neuronal signals were recorded during the third week of the culture. All the devices have successfully detected the electrophysiological signals of the cultured neurons.

For detailed characterization of the detection performance of different types of electrodes, we evaluated the number of active electrodes on each device using the Cerebus software from Blackrock Microsystems (Figure S6-1 A&B). It was found that the average number of active electrodes per device on the 3D-GMμEAs/PEDOT:PSS devices was 41.7 ± 1.8, which was significantly higher than that of the planar electrode devices (11.3 ± 0.9) (Figure S6-1 C).

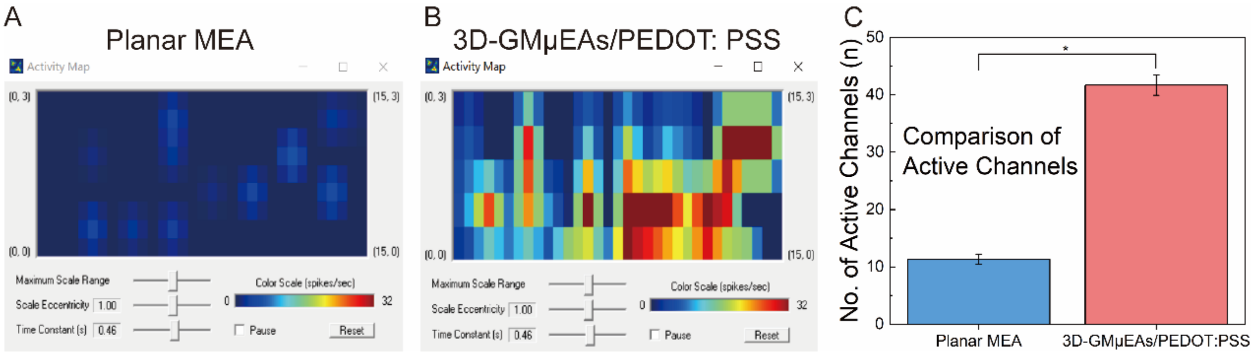

Figure S6-1. Comparison of active channels between 3D-GMμEAs/PEDOT:PSS and planar electrodes. (A) Activity map of a representative planar electrode device. (B) Activity map of a representative 3D-GMμEAs/PEDOT:PSS device. (C) Comparison of the average number of active channels between planar devices and 3D-GMμEAs/PEDOT:PSS devices (n=3, i.e. three independent devices per type, *p<0.05).

Next, we evaluated the amplitude and signal-to-noise ratio (SNR) of the neural signals detected by the devices. Data from randomly selected nine active channels from a representative device of each type were selected for further analysis, as shown in Figure S6-2. Within a 300s timeframe, signals from the majority of planar electrodes were in the range of ±100 μV (Figure S6-2 A), whereas those from the 3D-GMμEAs/PEDOT:PSS electrodes were in the range of ±200 μV (Figure S6-2 B). The peak-to-peak amplitude from the 3D-GMμEAs/PEDOT:PSS electrodes was 386.3 ± 21.9 μV compared to 175.7 ± 7.2 μV for the planar electrodes (Figure S6-3 C), and the SNR from the 3D-GMμEAs/PEDOT:PSS electrodes (13.8 ± 0.8) was also higher than that of planar electrodes (6.3 ± 0.3)(Figure S6-3 D).

Furthermore, statistical analysis of the firing timestamps of action potentials shows that the average firing rate detected by the 3D-GMμEAs/PEDOT:PSS electrodes (1.78 ± 0.40 Hz) is also significantly higher than the planar electrodes (0.48 ± 0.10 Hz)(Figure S6-3 E), indicating a higher sensitivity. Taken together, it is evident that the 3D-GMμEAs/PEDOT:PSS electrodes outperformed the planar electrodes significantly in all the tested metrics, elucidating their superior detection capabilities.

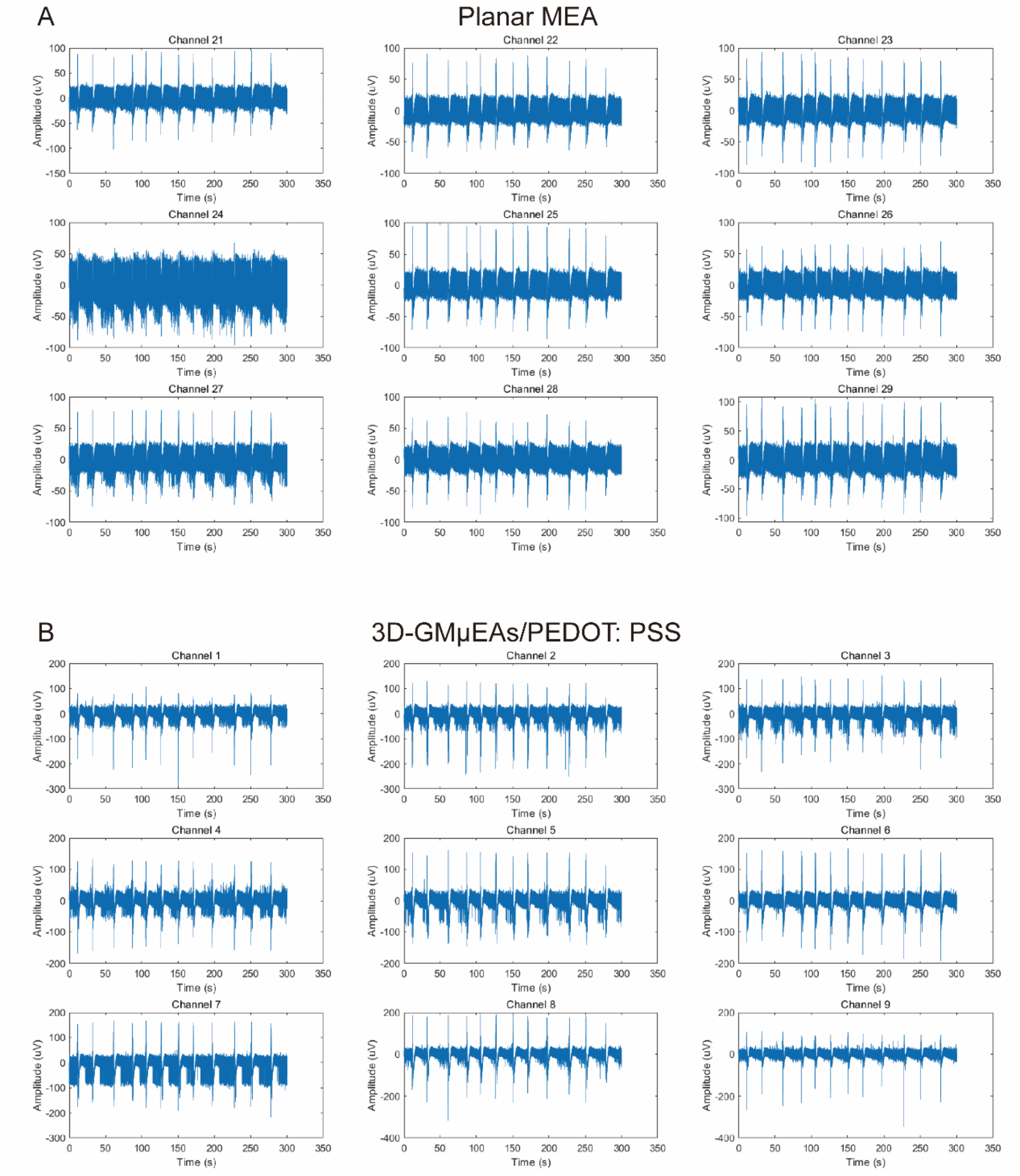

Figure S6-2. Raw data recordings from randomly selected nine channels (i.e. active electrodes) of a representative device of each type. (A) Planar electrodes. (B) 3D-GMμEAs/PEDOT:PSS electrodes. Neural Processing MATLAB Kit - NPMK was used to plot these raw data.

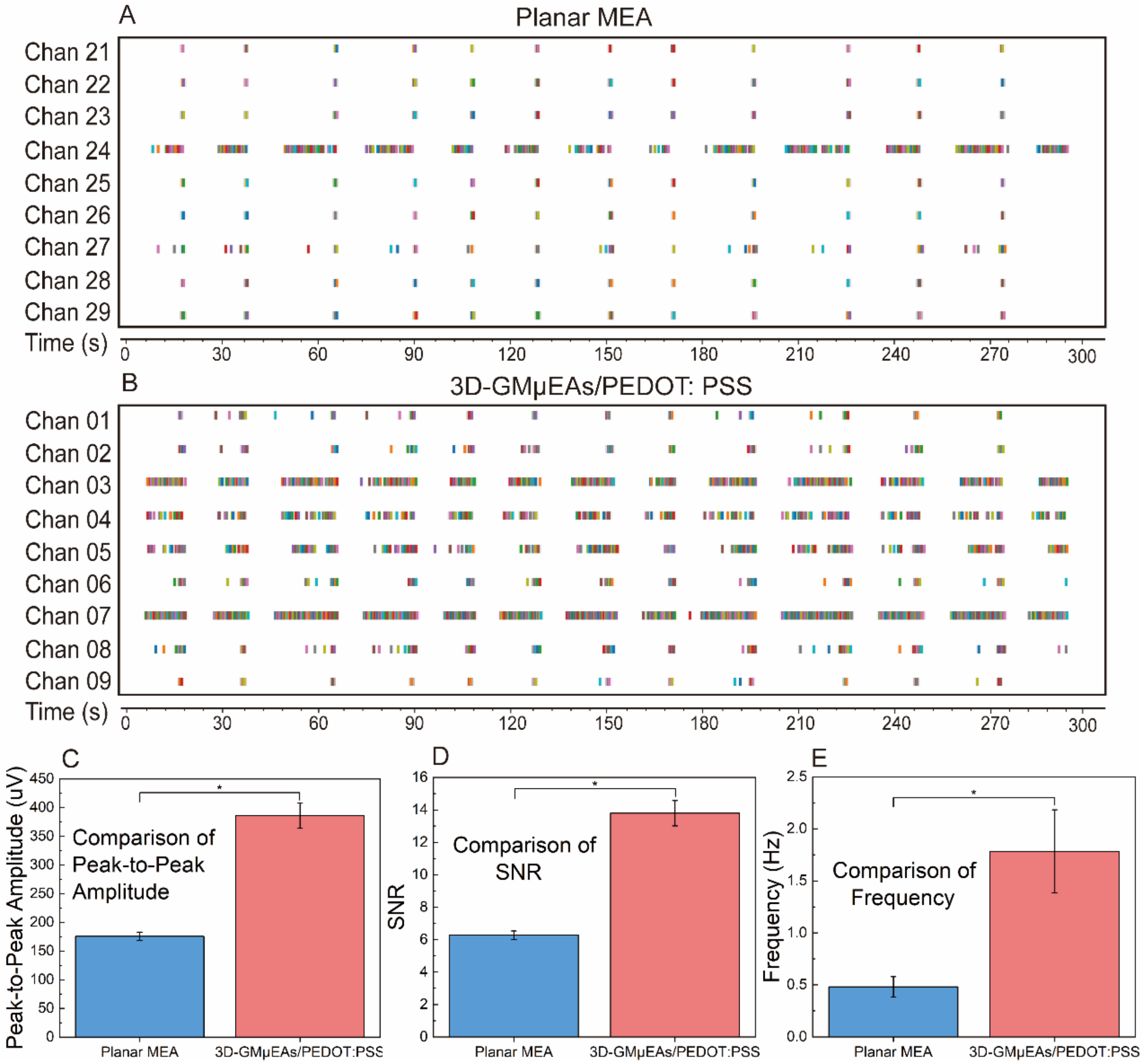

Figure S6-3. Comparison of detection performance between the planar and 3D-GMμEAs/PEDOT:PSS electrodes. Nine active electrodes channels were randomly selected from a representative device of each type. (A) Timestamps of spikes discharged within 300s by the planar electrodes. (B) Timestamps of spikes discharged within 300s by the 3D-GMμEAs/PEDOT:PSS electrodes. (C) Average peak-to-peak amplitude (*$p<0.05$). (D) Average signal-to-noise ratios (*$p<0.05$). (E) Average firing rates (*$p<0.05$).

S7 Characterization of neuronal network communication capability

In the field of neuroscience, assessing the functional connectivity of neuronal networks through correlation and mutual information between neurons is a well-established method. This approach is widely utilized to study interactions and communications among different brain regions and neurons. For instance, studies have employed correlation and mutual information to assess the functional connectivity of various neuronal networks and brain areas[1-3]. However, approaches for

quantitively evaluating the efficacy of these functional connectivity are lacking. To better characterize functional connectivity across different neuronal networks, especially during different developmental processes, we propose to quantify the dynamic changes in neuronal networks under different states through a measure of "neuronal network communication capability".

Specifically, we define "the neuronal network communication capability" using key metrics derived from the correlation heatmaps and mutual information networks, shown in Equation S7-1.

Neuronal Network Communication Capability = F(correlation heatmap metrics) + F(mutual information network metrics) Equation S7-1

Where the function F(correlation heatmap metrics) = α × average correlation coefficient. The correlation coefficient is used to statistically measure the linear relationship between two neurons, calculated through the Pearson correlation coefficient, as follows:

$$r = \frac{\sum(X_i - \bar{X})(Y_i - \bar{Y})}{\sqrt{\sum(X_i - \bar{X})^2(Y_i - \bar{Y})^2}} \quad \text{Equation S7-2}$$

where r is the Pearson correlation coefficient. X_i and Y_i are the values in the action potential time series, and $\bar{X}$ and $\bar{Y}$ are their mean values. In this study, the average correlation coefficient is the mean of correlation coefficients in the correlation matrix that are greater than 0.5.

The function for mutual information network metrics, **F(mutual information network metrics) = β × average mutual information + γ × average degree + δ × clustering coefficient.**

Where the "average mutual information" serves as a quantitative measure to assess the extent of information exchange between two neurons, elucidating the extent to which the activity state of one neuron informs about that of another. Based on the concept of entropy, mutual information captures both linear and nonlinear relationships between neurons, making it a generic tool for analyzing the functional connections of neuronal networks. The calculation formula is as follows:

$$MI(X;Y) = \sum_{x \in X} \sum_{y \in Y} p(x,y) \log\left(\frac{p(x,y)}{p(x)p(y)}\right) \quad \text{Equation S7-3}$$

where X and Y are two neuronal variables, $p(x,y)$ is the joint probability distribution of X and Y, and $p(x)$ and $p(y)$ are the marginal probability distributions of X and Y, respectively. A high mutual information value indicates a high degree of information sharing and interdependence among neurons. In this study, the average mutual information refers to the mean value derived from the mutual information matrix, wherein the mutual information exceeds the threshold of 0.1.

The "average degree" represents the average number of connections per node within the mutual information network, thereby serving as an indicator of the average connectivity density present within the neuronal network. Elevated values of the average degree typically imply a higher abundance of communication pathways among nodes, potentially indicating stronger communication efficacy and a more intricate network architecture.

The "clustering coefficient" measures the degree of clustering among nodes in the mutual information network. A higher clustering coefficient indicates a tendency for neurons to form tightly interconnected groups. Networks with a high clustering coefficient usually imply a more efficient information transfer, as closely connected groups of neurons can synchronize and relay signals more effectively. Such network structures play a pivotal role in reducing redundancy and optimizing efficiency in information transmission.

Our method provides a quantitative assessment of neuronal network communication capability by integrating correlation and mutual information metrics, each representing different aspects of neuronal connections and interactions. The coefficients α, β, γ, and δ in the formula are adjustable parameters, enabling customization to suit different models.

Building on the aforementioned content, we calculated various metrics for neurons across different days and after electrical stimulation, as shown in Figure S7. When the coefficients α, β, γ, and δ in the formula are all set to 1 (in this context, the communication capability of neuronal networks is considered only in terms of relative magnitude between states), we are able to determine the magnitude of neuronal network communication capability.

The results indicate (Figure S7) that on Day 7, due to the lack of established effective network connections, all statistical values are zero. Subsequently, from Day 14 onwards, connections in the mutual information network among neurons began to form, characterized by high correlation and mutual information among a small portion of neurons (Figure S7A). However, the clustering coefficient remains low (only at 1) (Figure S7B), indicating relatively lower communication capability within the neuronal network. By Day 21, both the clustering coefficient and average degree increased substantially (Figure S7 B&C), leading to a significant increase in the final neuronal network communication capability. In summary, as the neuronal network matures, its communication capability progressively amplifies.

Furthermore, upon subjecting the same neuronal network on Day 21 to electrical stimulation training, a further enhancement in neuronal network communication capability is observed, as illustrated in the increased values in the average correlation & the average mutual information (Figure S7A), the average degree &the clustering coefficient (Figure S7B), as well as communication capability and capability proportions (Figure S7C).

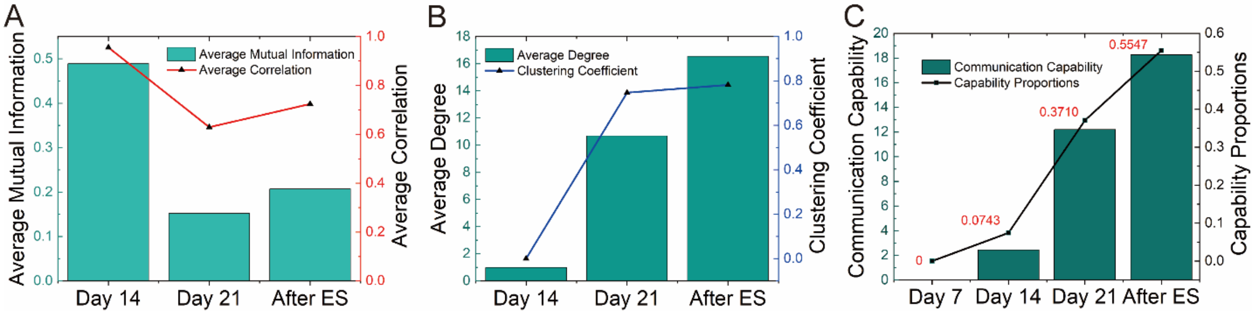

Figure S7. Assessment of neuronal network communication capability at different stages. (A) Statistics on average mutual information and average correlation. (B) Statistics on the clustering coefficient and average degree of mutual information networks. (C) Values and relative magnitudes of communication capability. Data are from all the active electrodes from a representative 3D-GMμEAs/PEDOT:PSS device.

S8 Analysis of spontaneous activity of neurons across days

Burst discharge refers to a series of synchronous events in which neurons in a network discharge and is typically closely associated with information transmission and neuronal network formation. With time, burst discharge becomes more frequent, indicating that the connection and activity of the neuronal network are gradually strengthening. Specifically, the frequency and size of bursts depend on the connectivity and excitability of neurons. As neuronal network connections and

excitability levels increase, neurons may fire bursts more frequently. This is often an important indicator of the maturity of a neuronal network, as it suggests that the connections between neurons are strengthening, and the network is gradually forming and optimizing. By analyzing the statistics of burst analysis on different days, Table S2, we concluded that the neuronal network was formed after 21 days.

Table S2 Statistics of bursts of neurons across days

Burst / Day	Num. Bursts	% of Spikes_in Bursts	Mean Burst_Duration	Mean Spikes_in Burst	Mean ISI_in Burst	Mean Freq._in Burst
7	2	3.3	0.14	4.50	0.04	25.66
14	39	5.5	0.13	5.20	0.03	38.40
21	634	36.7	0.15	7.93	0.02	67.27

S9 Calculation of network communication time

In the field of neuronal network research, the time required for communication between neurons is a crucial factor to consider. We define this time as the network communication time, which represents the time difference between the first and last neuron delivery in a network, provided that the difference between adjacent delivery times is not greater than the maximum synaptic latency (SL) time of 10 ms. When the first neuron in a network fires, it communicates with other neurons in the network. We have demonstrated that SL is evidence of network communication but delays greater than 10 ms may result from independent neurons firing spontaneously. Thus, we removed such data to accurately calculate the overall network communication time. Our statistical analysis of network communication speed is based on this metric, which provides insight into the speed at which neuronal networks communicate and presents a valuable index for studying neuronal network dynamics.

In this study, we investigated the impact of electrical stimulation on neural activity and network communication. Supplementary Figure S9A-C displays the complete time series of a burst before and after electrical stimulation. We observed that the overall discharge time during electrical stimulation was shorter than that after stimulation and shorter than that before stimulation. This finding indicates that electrical stimulation alters the SL time between neurons, resulting in changes in correlation between neurons and, in turn, changes in the functional connection network.

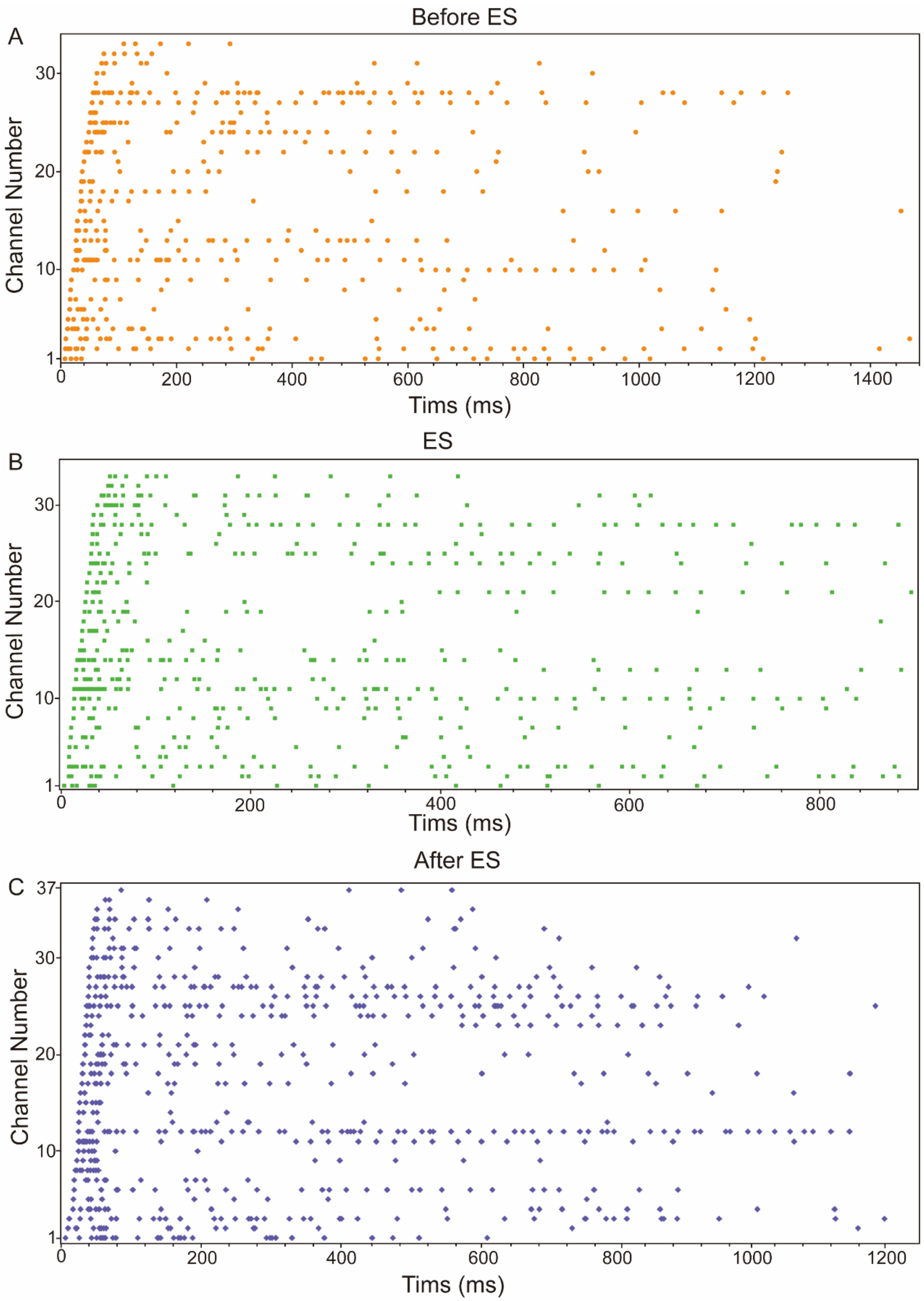

Figure S9 Time series of entire spikes in a burst of neurons. (A) Before electrical stimulation (ES), (B) During ES. (C) After ES.

S10 Calculation of network communication speed

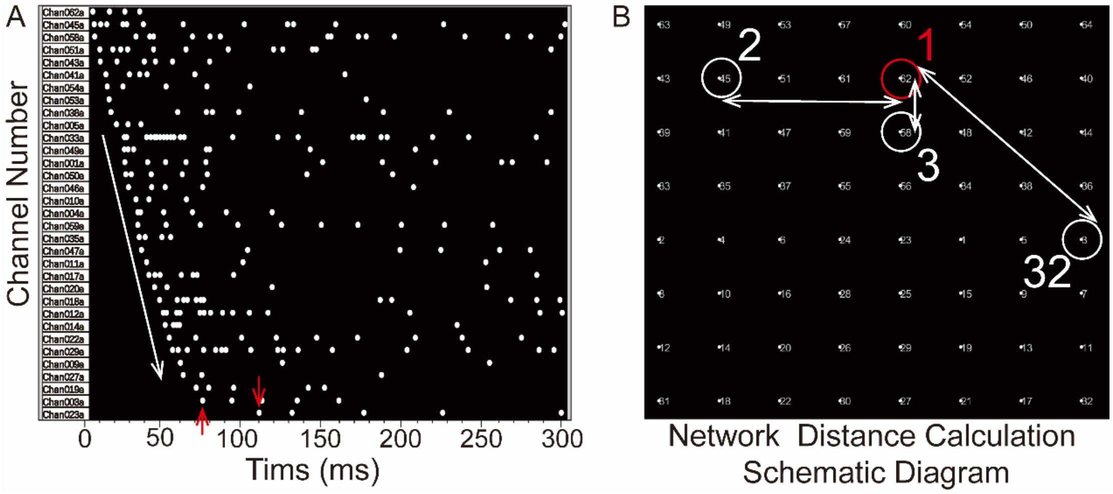

Figure S10-1: Calculation of network communication speed. (A) Schematic illustration of the method for calculating network communication time. The white arrow indicates the propagation direction of the neural signal, and the two red arrows mark the time points beyond the maximum SL rules. (B) Calculation diagram of network distance. The first discharge site was selected as the central point, marked in red as "1" in panel B, and the distance from site 1 to the corresponding site of each discharge time point meeting the requirements was calculated.

Based on the calculation of network communication time, the distance of network communication was further estimated, and the network communication speed was determined by finding the slope of the fitting line. Firstly, the first firing site was selected as the central point, as shown in Figure S10-1AB site 62, marked as red 1 in Figure S10-1B. The distance from 1 to the corresponding site of each discharge time point that meets the requirements was calculated, for example, the second point 45, marked as white 2 in Figure S10-1B. By following this approach for other sites, the dynamic starting point was calculated using MATLAB.

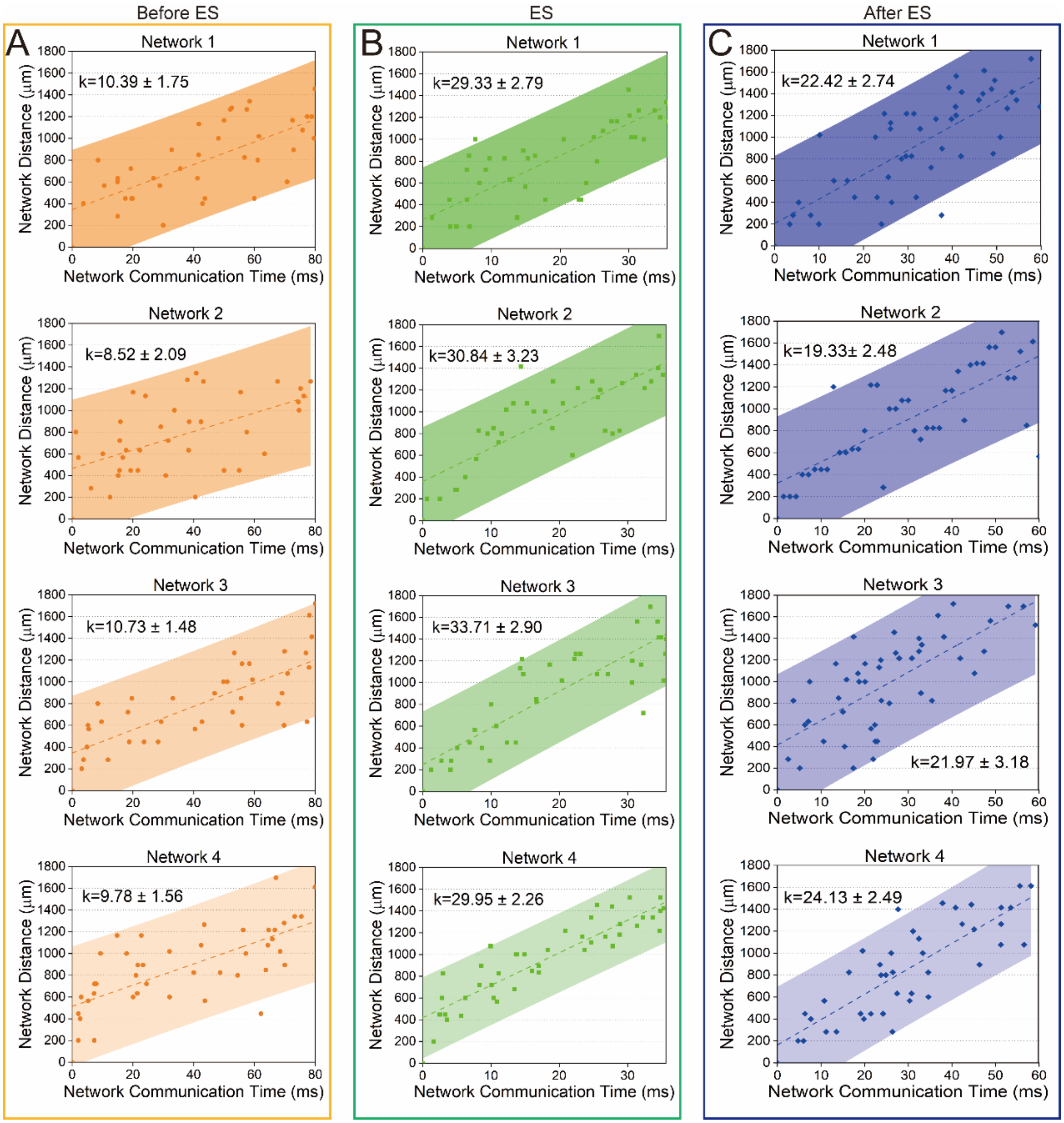

Figure S10-2. Scatter plot of network distance to all firing sites from the first firing site depending on network communication time before (A), during (B), and after electrical stimulation (C). The dotted line and shaded area represent the best fit of linear regression and the 95% confidence level, respectively. The slope of the linear regression denotes the network communication velocity.

S11 Calculation of synaptic delay across days

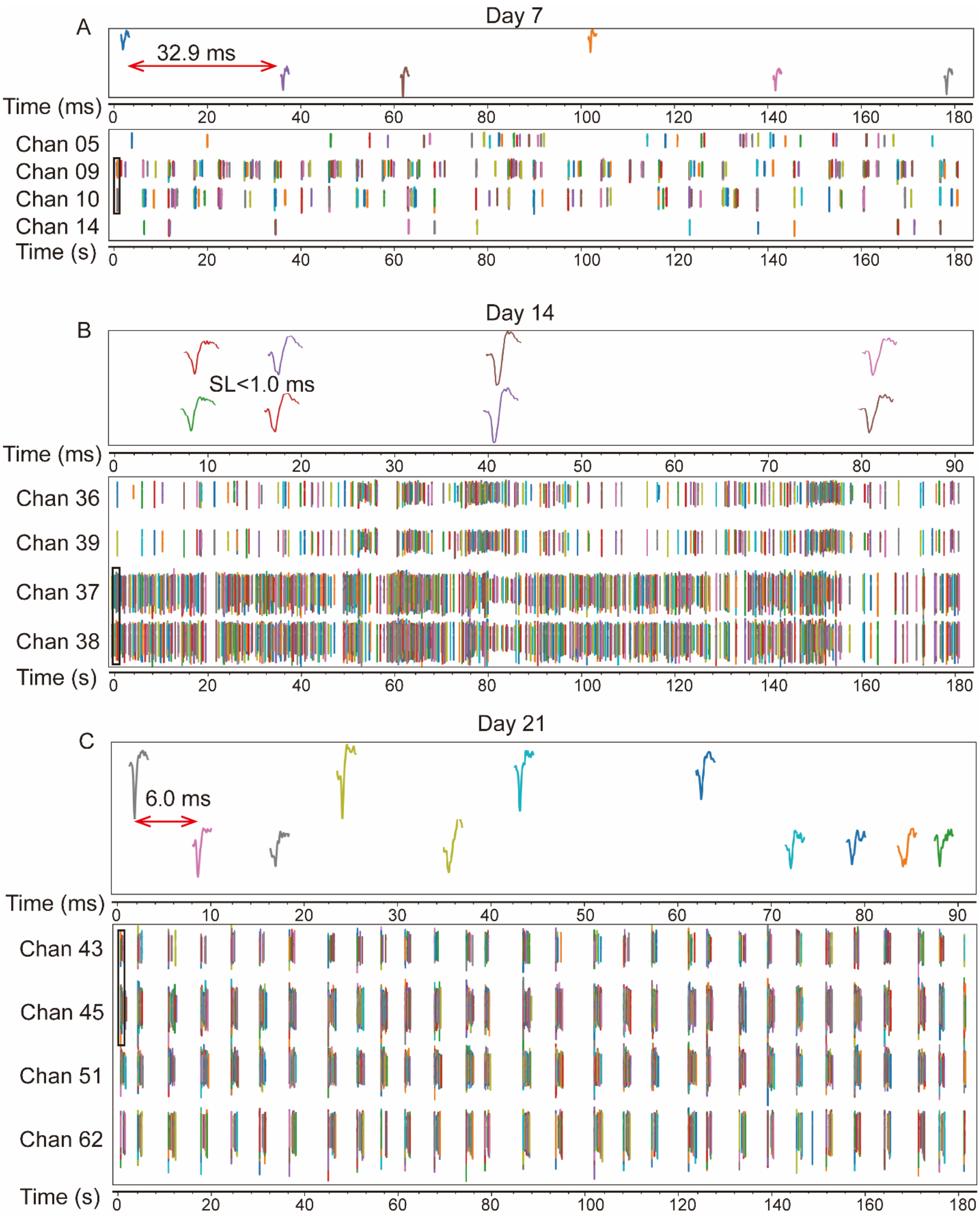

Figure S11-1. Calculation of synaptic delay across days. (A) day 7. (B) day 14 (C) day 21.

We analyzed spontaneous discharges of neurons on different days in vitro culture and focused on calculating the synaptic latency (SL) of mutual neurons. Supplementary Figure S11-1A and S11-2A demonstrates that on the seventh day, the delay time of synaptic information transmission between neurons is significantly longer than the time of normal synaptic transmission, indicating that there is no connection between neurons, at this time. On day 14, the SL time is less than 1ms, suggesting a direct connection between two neurons and explaining the strong connection in the mutual information network on this day, Figure S11-1B, S11-2B, and Figure 6E. For Day 21, we observed complex network connections and calculated an average SL of approximately 6ms, which is considered a normal SL time, Figure S11-1C, S11-2C, and Figure 6F.

Our results suggest that the delay time of synaptic information transmission plays a crucial role in determining the connectivity between neurons. Longer SL times may indicate no connection between neurons, while shorter delay times suggest direct connections. The complex network connections observed on day 21 may reflect a combination of direct and indirect connections between neurons. Our findings provide insights into the dynamics of neuronal networks and the factors that contribute to the formation of functional connections.

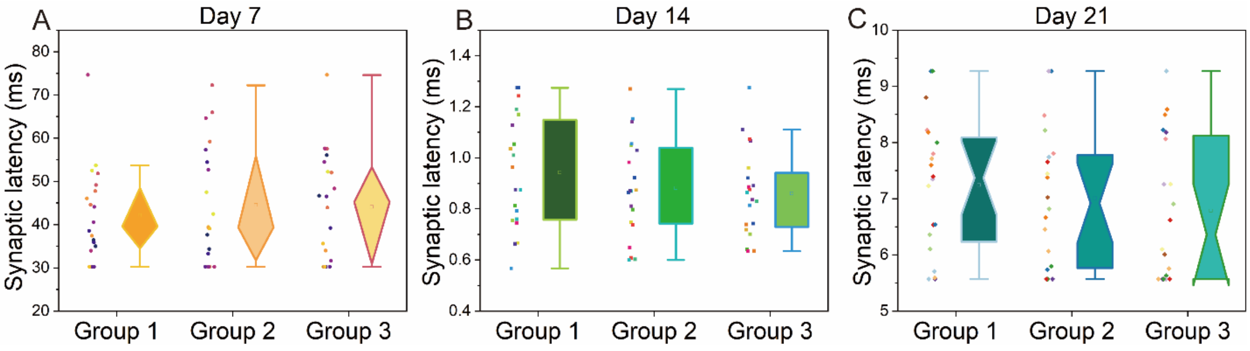

Figure S11-2. Data distribution of synaptic delay times for Figure 7H.

S12 Explanation of the neuronal network communication connectivity model

Here, we delve into the neuronal network communication connectivity model, focusing on elucidating the differences between network connections and synaptic connections. Neuronal networks exhibit distinct connection states at different developmental stages (based on physical synaptic connections). These diverse connection patterns lead to different functional connections. At the microscopic level, this is manifested as variations in synaptic delay or network communication speed, which underlies functional connections. At the macroscopic level, it is reflected in differing network communication capacities. Therefore, the communication connectivity model unveils the intricate relationships between neuronal network connections and function. It is imperative to introduce the hierarchical structure of neuronal networks in these representations, as a network's representation encapsulates the entirety of relationships between neurons.

Below, we delineate the disparity between the representation of neuronal networks and the representation based on the relationships between neurons:

Relationship between network communication connectivity and synaptic connections:

Synaptic connections, fundamental in neuroscience, describe the physical linkage between two neurons. Each synaptic connection serves as a basic unit of information transfer, governing signal transmission between neurons. Network communication connectivity describes the structural characteristics of information transfer across the entire network, amalgamating of multiple synaptic connections. It encompasses potential communication pathways among all neurons, extending beyond direct physical connections between individual neurons.

Relationships between network communication capability and synaptic strength:

Synaptic strength refers to the efficiency or strength of a single synaptic connection in signal transmission, quantifiable by the postsynaptic potential magnitude. Network communication capability is related to the efficiency of information transfer across the entire network holistically, incorporating contributions from individual synapses and the collective effects of multiple pathways and cascading communications within the network.

In summary, while synaptic connections and strength are key indicators of interactions between individual neurons, our concepts of "network communication connectivity" and "network communication capability" offer a comprehensive and macroscopic viewpoint for exploring how neuronal networks collaboratively process and transmit information. These concepts facilitate the comprehension of network dynamics and functional structures beyond the individual synapse level, providing important tools and frameworks for understanding the brain's holistic functionality.

S13 Fabrication of the 3D-GMμEAs/PEDOT:PSS

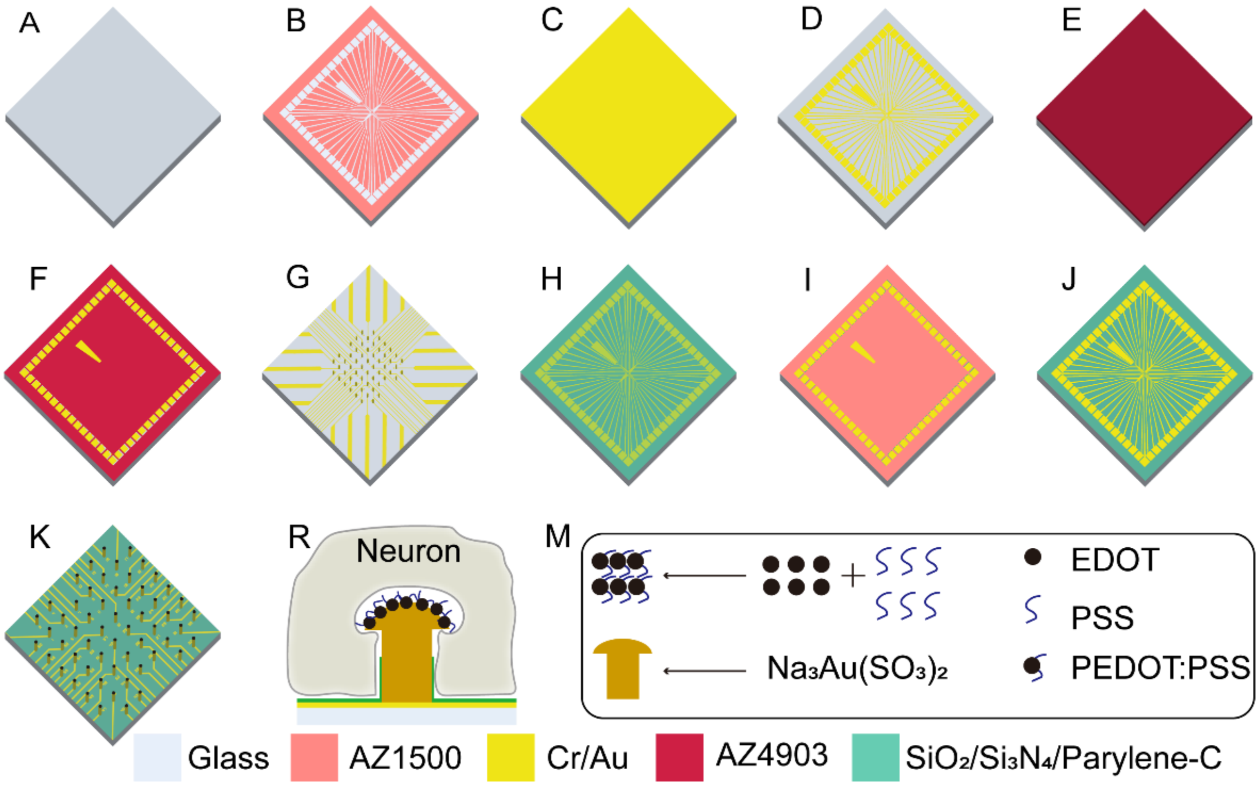

Figure S13. Fabrication and modification of the 3D-GMμEAs/PEDOT:PSS. (A) Substrate cleaning process. (B) Patterning of microelectrodes, wires, and contact pads on the substrate using the first photolithography. (C) Sputtering of a Cr/Au conductive layer. (D) Lift-off process. (E) Coating of the substrate with a thick layer of a positive photoresist (AZ4903). (F) Lithography and development process to access the electrode sites. (G) Gold electroplating in a gold bath, followed by the removal of the photoresist to expose the GMμEs. (H) Deposition of an insulation layer. (I-J) Steps to create openings in the insulation layer. (K) Deposition of PEDOT:PSS onto the GMμEs. (R) Schematic illustration showing that GMμEs modified with PEDOT:PSS have good coupling with neurons. (M) Modified materials.

Figure S13A-D details the process flow for fabricating flat electrodes with a pattern of conductive material on a quartz glass substrate. The process begins with cleaning the substrate using acetone, isopropyl alcohol, and deionized water, followed by boiling in concentrated sulfuric acid for 30 minutes and drying in an oven, Figure S13A. Next, photoresist AZ1500 is spin-coated onto the substrate, exposed through a mask plate to define the electrode pattern layout, and developed, Figure S13B. Then, a conductive layer is sputtered onto the substrate, starting with a 30 nm Cr adhesion layer and followed by a 200 nm Au electrode layer, Figure S13C. Finally, the photoresist is removed in acetone to reveal a flat electrode with a pattern of conductive material, Figure S13D.

Figure S13H-J depicts the process flow for fabricating gold mushroom electrodes with high sidewall insulation on a silicon oxide and silicon nitride substrate. The first step involves depositing a 300 nm layer of silicon oxide and a 500 nm layer of silicon nitride using plasma-enhanced chemical vapor deposition (PECVD), optionally followed by coating with parylene-C

for higher sidewall insulation, Figure S13H. Next, a second mask plate is used to open the surface area of the gold mushroom electrode and the external contact pad, similar to the steps in Figure S13B. The exposed electrodes, wiring, and contact pads are then etched using CHF3 reactive ion etching, Figure S13J. Finally, a glass ring is glued to the surface of the electrode with silicone to provide a container for neuron culture (Figure 2A).

S14 Fabrication of GMμEs.

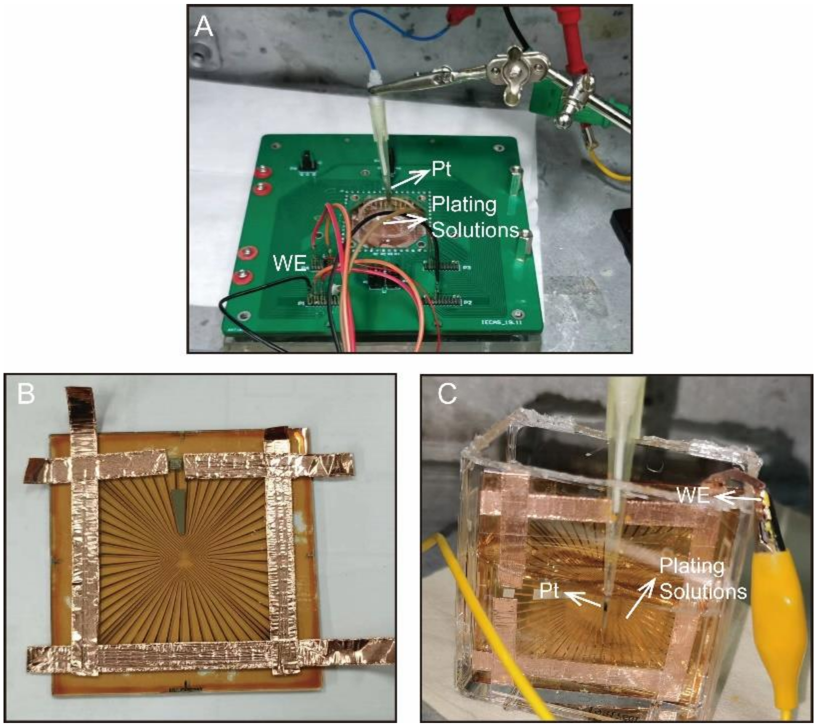

Figure S14. Electroplating methods for GMμEs. (A) Circuit interface boards for selective plating of specific sites on the MEA. (B) External pads on the MEA are connected using conductive tape for one-shot plating. (C) Two-electrode system used for electroplating the gold mushroom electrodes.

We utilized two electroplating methods for the fabrication of GMμEs: selective plating and one-shot plating. Selective plating allows for targeted plating of specific sites, which can be achieved using the interface plate shown in Figure S14A. While this method enables customization and modification of individual electrodes, its efficiency is limited when only a few parts are electroplated due to the long electroplating time (~1 hour). To overcome this limitation, we employed the one-shot plating method, where we short-circuited the external pads of the electrodes using conductive tape, Figure S14B, connecting them together and so making a single working electrode by using a common wire during gold electrodeposition (Figure S14C). This approach allows for more efficient electroplating of multiple parts simultaneously.

S15 The effect of electroplating parameters on electrode height and electroplating stability

This section elucidates the relationship between electroplating parameters and electrode height, along with an explanation of electroplating stability. Here, we used the timed current method for electroplating, at a constant voltage of -1.5 V. Only electroplating time was adjusted while the other conditions maintained constant.

Regarding the electrode height, two scenarios were considered:

1) When the electrode height did not exceed the depth of the holes in the developed photoresist, as shown in Figures S15-1 (a-c), the surface area of the electrode remained constant during electroplating.
2) When the electrode height surpassed the depth of these holes, as illustrated in Figure S15-1 (d), continuous deposition led to a change in the electroplated electrode's surface area.

These two scenarios were discussed separately.

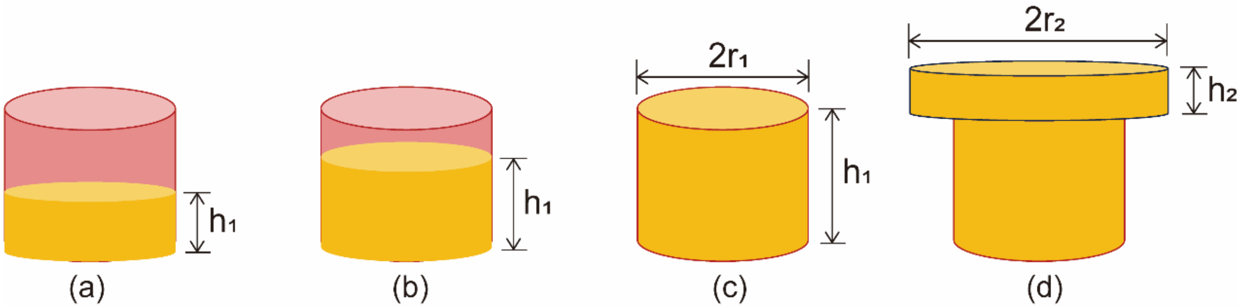

Figure S15-1. Schematic illustration of electrode changes during the electroplating process.

In this study, the formation of the three-dimensional structure primarily followed the first scenario. Electroplating data with different durations (Figure S15-2) were selected as model data to theoretically predict the relationship between electroplating time and electrode height. The algorithm is described as follows:

Firstly, the total charge of the electroplating process is calculated using the i-t curve, with the formula:

$$Q = \sum_{i=1}^{N-1} I_i \times \Delta t_i \qquad \text{Equation S15-1}$$

where I_i represents the current at each time point and Δt_i is the interval between two consecutive time points.

Then, according to Faraday's laws of electrolysis, the mass of the deposited metal can be calculated using the formula:

$$m = \frac{Q \times M}{n \times F} \qquad \text{Equation S15-2}$$

where m is the mass of the deposited metal, Q is the total charge, M is the molar mass of the metal (for gold, 196.97 g/mol), n is the valency of the metal, and F is Faraday's constant.

Finally, the height of the deposit is calculated using the formula:

$$h = \frac{m}{\rho \times A} \qquad \text{Equation S15-3}$$

where h is the height of the deposit, ρ is the density of the metal, and A is the surface area of the electrode.

Based on the theoretical calculation, the electrode heights under four electroplating durations were approximated (Figure S15-2E). The results suggested that an electroplating duration of 4000s would yield three-dimensional micropillars approximately 30 μm in height, which matched well with the experimental data. Therefore, an electroplating time of 4000s was used for the formation of three-dimensional structures.

To verify the stability of the electroplating effect under this parameter, the radius of a series of electrodes electroplated under these conditions was measured using a microscope (Figure S15-3A). Statistical analysis of these values indicated that the radius of the electrodes under these parameters was 15.19 ± 0.10 μm (Figure S15-2F), demonstrating that the electroplating process was stable and controllable.

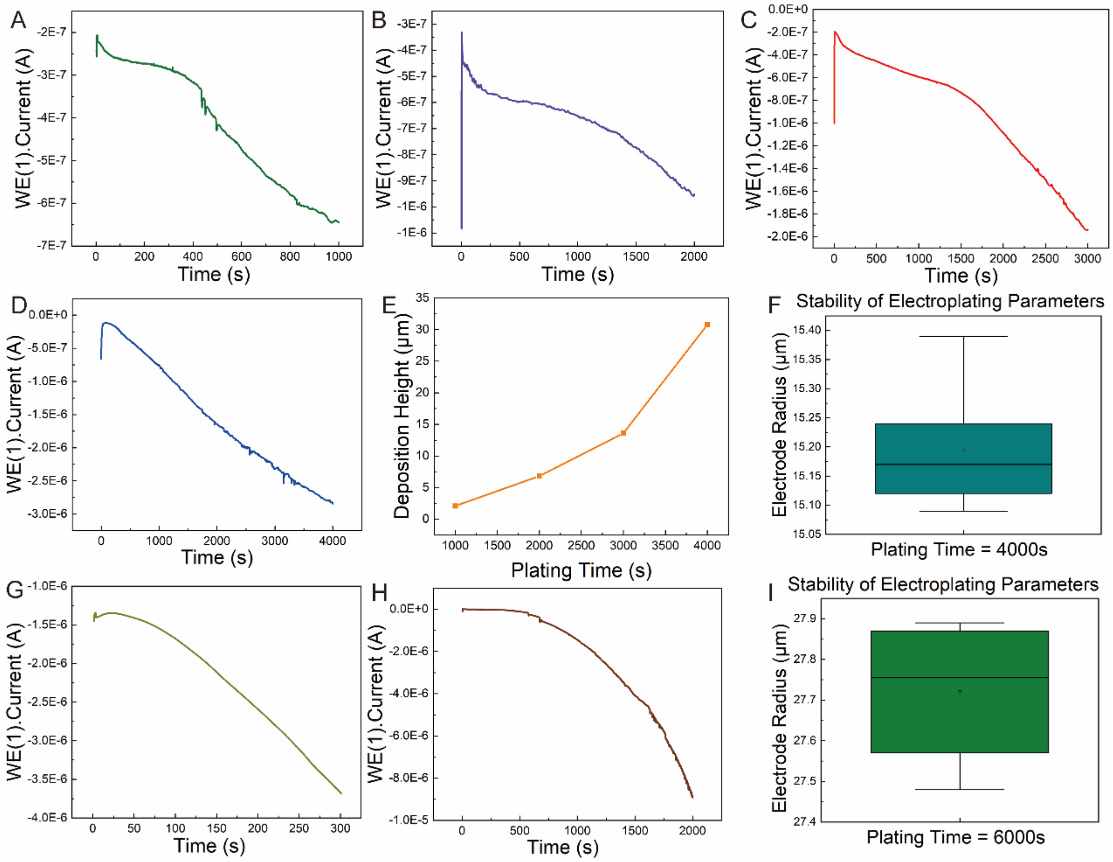

Figure S15-2. The effect of electroplating parameters on electroplating height and electroplating stability. (A) i-t curve for electroplating duration of 1000s. (B) i-t curve for electroplating duration of 2000s. (C) i-t curve for electroplating duration of 3000s. (D) i-t curve for electroplating duration of 4000s. (E) Relationship between electroplating time and electrode height. (F) Box plot of electrode radius for electroplating time of 4000s. (G) i-t curve for an additional 300s of electroplating after completing scenario 1. (H) i-t curve for an additional 2000s of electroplating after completing scenario 1. (I) Box plot of electrode radius for electroplating time of 6000s.

In the second scenario (as shown in Figure S15-1(d)), predicting the relationship between electroplating time and dimensions becomes more complex due to the change in the electrode's surface area during electroplating. Moreover, given the difficulty in accurately measuring several key variables, we are compelled to rely on certain assumptions to establish this relationship. These assumptions may introduce some discrepancies between our simulation results and the actual electroplating process. Nevertheless, this predictive model still holds significant value as it provides a reference for optimizing the electroplating process. For the second phase, we chose the i-t curve corresponding to an electroplating duration of 300s (which is the time for subsequent electroplating after completing the first scenario) as the basis for calculation. Initially, we calculate the total charge *Q* from the data file, then determine the added mass *m*, and subsequently compute the volume of the deposited metal V = m/ρ. To estimate the approximate change in electrode dimensions during this phase, we assume a uniform distribution of the deposited metal both laterally and longitudinally, forming a regular cylinder. We assume that the increase in the cylinder's radius is equal to the height increase: $r_2 - r_1 = h_2$. Using the formula for the volume of a cylinder, the height h_2, and thus the increase in the radius, can be calculated.

Applying this algorithm to the data from Figure S15-2G, an increase in height and radius of 2.24 μm was obtained. Assuming linear growth during electroplating, the radius increase rate is approximately 0.00746 μm/s. It is important to note that this parameter serves only as a reference, providing an approximate electroplating time necessary for customizing electrode sizes. Using this result, we estimated that an increase of 15 μm in the electrode radius would require approximately 2000 seconds of electroplating time. Based on this estimation, we prolonged electroplating for an additional 2000s after completing the first scenario (as shown in Figure S15-2H). The average radius of a series of electrodes electroplated under these conditions is 27.72 ± 0.19 μm (Figure S15-2I, Figure S15-3B). While this indicates a slight deviation from our predicted radius of 30 μm, it underscores the utility of our parameter recommendations and reaffirms the consistency of electroplating under these parameters.

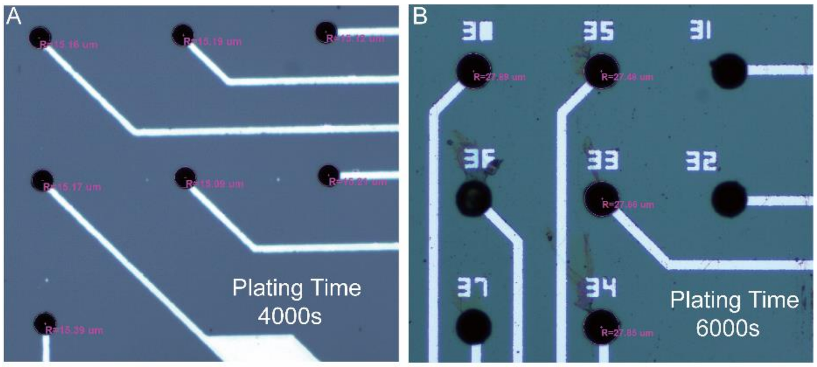

Figure S15-3. Characterization of electroplating parameter stability. (A) Radius of the electrodes after 4000s of electroplating. (B) Radius of electrodes after 6000s of electroplating. Radius of the electrodes were measured from their optical images.

S16 Process of electroplating PEDOT:PSS

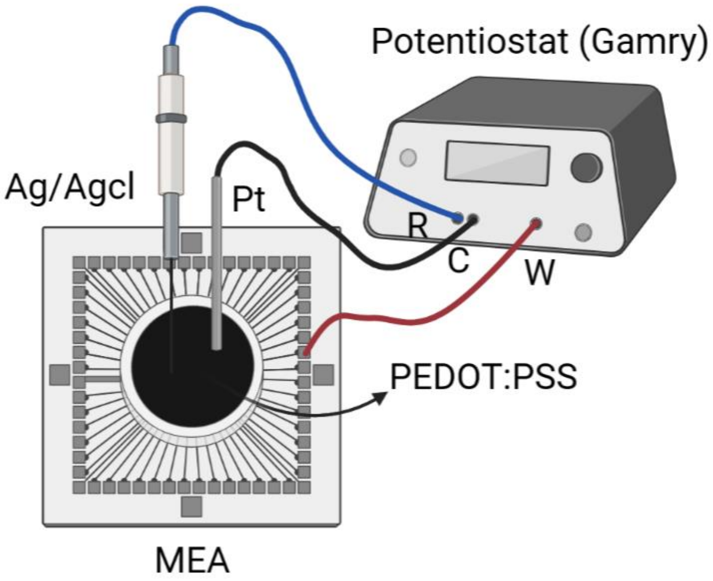

Figure S16. Three-electrode system electroplating PEDOT:PSS

We utilized a three-electrode system to modify PEDOT:PSS on GMμEs, as illustrated in Figure S16. The system consisted of a working electrode, a reference electrode, and a counter electrode. The plating solution employed in this study was PEDOT:PSS and the GMμEs served as the working electrode. The reference electrode was a reference point for measuring and controlling the potential of the working electrode without allowing any current to pass through it. To maintain a constant electrochemical potential at low current density, we employed Ag|AgCl as the reference electrode. The counter electrode, or auxiliary electrode, formed a circuit with the working electrode to facilitate the current flow, thereby enabling the modification process to take place on the working electrode. Here, we utilized Pt as the counter electrode.

S17 Neuronal recording and electrical stimulation

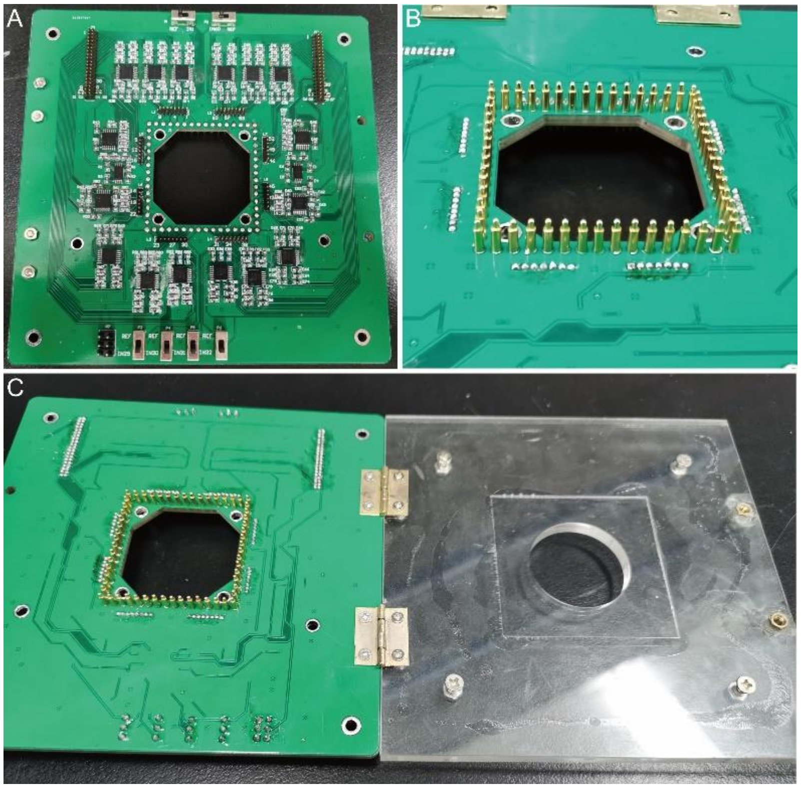

Figure S17. The circuit interface of MEA. (A) Circuit interface module with soldered connections. (B) Metal probes located on the back of the module. (C) The interface module is connected to the acrylic tray.

The interface module for detecting neuronal discharge information mainly consists of an acrylic MEA tray and a PCB detection interface module, with the PCB interface board designed using Altium Designer18 and the physical appearance after soldering shown in Figure S17A. The back structure of the PCB board is shown in Figure S17B, where the metal spring pins are used to fix the MEA and transmit the neural signal. The acrylic tray is designed to hold the MEA in the middle, with a through-hole in the middle for observing the status of the MEA, as shown in Figure S17C. At the same time, an external electrical stimulation interface is designed on the PCB circuit board, allowing the module to measure neuronal discharge during drug regulation and test the discharge of neuronal electrophysiological signals under external electrical stimulation. Considering the compatibility of the PCB interface detection module with various microelectrode arrays, this design has six out of 64 channels designed as toggle switches, which can be used as both reference and working electrodes.

(1) So, K.; Koralek, A. C.; Ganguly, K.; Gastpar, M. C.; Carmena, J. M. Assessing Functional Connectivity of Neural Ensembles Using Directed Information. *Journal of Neural Engineering.* **2012**, *9* (2).

(2) Cohen, M. R.; Kohn, A. Measuring and Interpreting Neuronal Correlations. *Nature Neuroscience.* **2011**, *14* (7), 811-819.

(3) Varley, T. F.; Pope, M.; Faskowitz, J.; Sporns, O. Multivariate Information Theory Uncovers Synergistic Subsystems of the Human Cerebral Cortex. *Communications Biology.* **2023**, *6* (1).
